# Methylation-associated silencing of microRNA-129-3p promotes epithelial-mesenchymal transition, invasion and metastasis of hepatocelluar cancer by targeting Aurora-A

**DOI:** 10.18632/oncotarget.12870

**Published:** 2016-10-25

**Authors:** Shiyun Cui, Kai Zhang, Chen Li, Jing Chen, Yan Pan, Bing Feng, Lei Lu, Ziman Zhu, Rui Wang, Longbang Chen

**Affiliations:** ^1^ Department of Medical Oncology, Jinling Hospital, School of Medicine, Nanjing University, Nanjing 210002, Jiangsu, PR China; ^2^ Liver Disease Center of PLA, The 81th Hospital of PLA, Nanjing 210002, Jiangsu, PR China; ^3^ Department of Hepatobiliary Surgery, First Hospital Affiliated to the Chinese PLA General Hospital, Haidian District, Beijing 100048, PR China

**Keywords:** hepatocelluar cancer, miR-129-3p, Aurora-A, epithelial-mesenchymal transition, metastasis

## Abstract

Metastasis and recurrence has become one major obstacle for further improving the survival of hepatocelluar cancer (HCC) patients. Therefore, it is critical to elucidate the mechanisms involved in HCC metastasis. This study aimed to investigate the roles of microRNA (miR)-129-3p in HCC metastasis and its possible molecular mechanisms. By using microarray analysis to compare levels of different miRNAs in HCC tissues with or without lymph node metastasis (LNM), we showed that HCC tissues with LNM had reduced levels of miR-129-3p, which was related to its promoter hypermethylation and correlated with tumor metastasis, recurrence and poor prognosis. Gain - and loss - of - function assays indicated that re-expression of miR-129-3p could reverse epithelial-mesenchymal transition (EMT), and reduce *in vitro* invasion and *in vivo* metastasis of HCC cells. Aurora-A, a serine/threonine protein kinase, was identified as a direct target of miR-129-3p. Knockdown of Aurora-A phenocopied the effect of miR-129-3p overexpression on HCC metastasis. In addition, Aurora-A upregulation could partially rescue the effect of miR-129-3p. We further demonstrated that activation of PI3K/Akt and p38-MAPK signalings were involved in miR-129-3p-mediated HCC metastasis. These findings suggest that methylation-mediated miR-129-3p downregulation promotes EMT, *in vitro* invasion and *in vivo* metastasis of HCC cells via activation of PI3K/Akt and p38-MAPK signalings partially by targeting Aurora-A. Therefore, miR-129-3p may be a novel prognostic biomarker and potential therapeutic target for HCC.

## INTRODUCTION

Hepatocelluar cancer (HCC) is one of the most common malignant tumors around the world [1]. Although much progress made in early detection and adjuvant therapy, the overall survival of HCC patients still remains poor, which is partially caused by the high invasive potential and distant metastasis [2]. It is believed that elucidation of the mechanisms underlying HCC metastasis is crucial for exploiting novel therapeutic strategies in HCC.

MiRNAs are a class of small noncoding RNAs which negatively regulate gene expression at post-transcription level through their base-pairing with the 3′-untranslated region [3]. Accumulating evidence indicates that miRNAs play important roles in a variety of celluar processes such as growth, differentiation, apoptosis and metastasis [4–6]. Many studies have pointed to the roles of deregulated miRNAs in tumor pathogenesis by targeting oncogenes or tumor suppressors [7]. Recently, sets of miRNAs in HCC by high-throughput screening can be used to predict prognosis or make early diagnosis of this disease [8, 9]. Also, some reports have focused on the effects of miRNAs in the metastatic process of HCC cells. For example, miR-140-5p was reported to suppress HCC growth and metastasis by targeting TGFB1 and FGF9 [10]. Yang’ et al reported that miR-26a suppresses HCC growth and metastasis by regulation of interleukin-6-Stat3 pathway [11]. The same research group reported that miR-26a suppresses HCC angiogenesis by targeting HGF/c-met signaling [12]. Of particular importance, miR-612 was found to be involved in from the initial to final steps of the metastatic cascade [13]. Meanwhile, other miRNAs, including miR-9 and miR-491, were reported to promote metastasis of HCC cells [14, 15]. Although several miRNAs with pro- or antimetastatic functions have been identified in HCC, whether other dysregulated miRNAs involved in HCC metastasis needs to be investigated. Therefore, elucidating the biological functions of miRNA dysregulation and identifying their targets will contribute to understanding the values of the miRNA-related signaling pathways so as to exploit miRNA-targeted therapeutic approach against HCC.

Here, by identifying a set of differentially expressed miRNAs between HCC tissues with or without LNM, we showed that miR-129-3p is significantly downregulated in metastatic HCC tissues and highly metastatic HCC cells. Reduced miR-129-3p in HCC is related to its promoter hypermethylation, and re-expression of miR-129-3p reverses EMT, inhibits *in vitro* invasion and *in vivo* metastasis of HCC cells at least partially by targeting Aurora-A, which finally induces the inactivation of PI3K/Akt and p38-MAPK signaling pathways. Thus, miR-129-3p will be a promising therapeutic target for the treatment of metastatic HCC.

## RESULTS

### Methylation-mediated miR-129-3p downregulation correlates with tumor metastasis and poor prognosis of HCC patients

**Figure 1:**
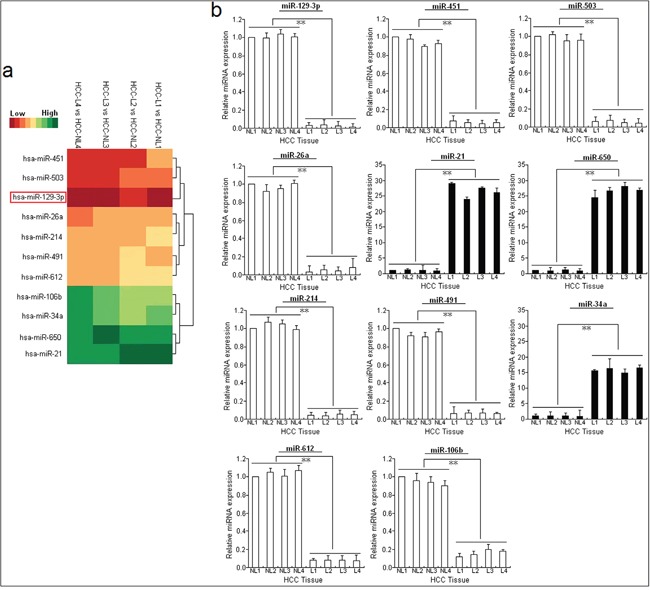
miR-129-3p is significantly downregulated in metastatic HCC cell lines or tissues **A.** MiRNA array analysis showed differentially expressed miRNAs in HCC tissues with LNM (HCC-L, n=4) and HCC tissues without LNM (HCC-NL, n=4). Overexpression is indicated in green, whereas underexpression is indicated in red. **B.** Validation of microarray analysis data by qRT-PCR. The relative expression levels of 11 dysregulated miRNAs (miR-129-3p, 451, 503, 26a, 21, 650, 214, 491, 34a, 612 and 106b) were determined by qRT-PCR. U6 was used as an internal control. Each qRT-PCR experiment was performed in triplicate.^*^P<0.05; ^**^P<0.01. HCC-NL: HCC without LNM; HCC-L: HCC with LNM.

**Figure 2:**
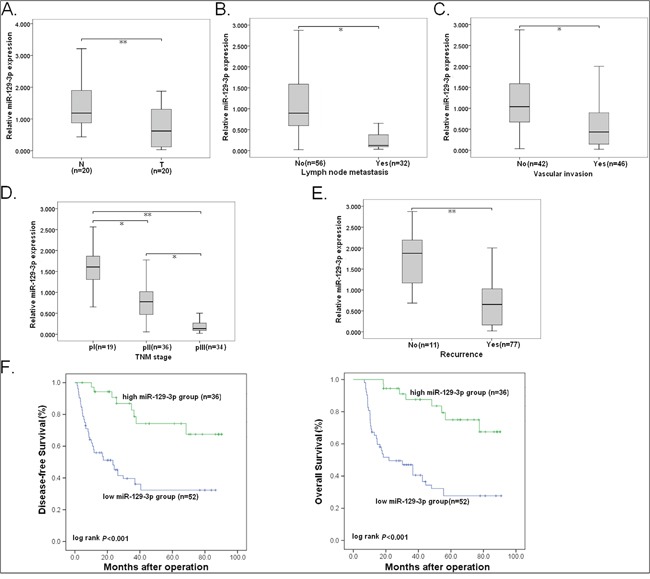
Reduced miR-129-3p correlates with HCC progression Expression of miR-129-3p was determined by qRT-PCR. U6 was used as an internal control. **A.** Relative miR-129-3p expression in paired HCC and adjacent nontumor liver tissues. T: HCC tissues; N: nontumor liver tisssues. **B.** Relative miR-129-3p expression in HCC tissues with or without LNM. **C.** Relative miR-129-3p expression in HCC tissues with or without vascular invasion. **D.** Relative miR-129-3p expression in HCC tissues with different TNM stage. **E.** Relative miR-129-3p expression in HCC tissues with or without recurrence. **F.** Kaplan-Meier analysis of DFS or OS of HCC patients. The survival data were compared with the log-rank test. Each experiment was performed in triplicate. ^*^P<0.05; ^**^P<0.01.

To identify the dysregulated miRNAs with HCC metastasis, we comparatively analyzed the miRNA profiles in HCC tissues with (n=4) and without (n=4) LNM using a microarray platform. Compared with tissues without LNM, HCC tissues with LNM showed 58 dysregulated miRNAs (fold change >2.0), among which 24 were significantly downregulated and the other 34 were upregulated (Table 1). A heat map of these 11 qRT-PCR-verified miRNAs (fold change >10.0) was generated based on the relative miRNA expression (Figure 1A). Using TaqMan qRT-PCR, we confirmed that the relative expression levels of those 11 dysregulated miRNAs were consistent with the microarray data, and miR-129-3p was the most common downregulated miRNA in HCC samples with LNM (Figure 1B). Then, the expression of miR-129-3p in 20 paired of HCC and adjacent nontumor tissues was detected. It was observed that miR-129-3p was significantly downregulated in HCC tissues (Figure 2A), which prompts us to further investigate the clinical significance of miR-129-3p in HCC development by extending our miR-129-3p quantification assay to a cohort of 88 HCC tissues.

**Table 1:** Differentially expressed miRNAs in HCC tissues with LNM as compared to HCC tissues without LNM

miRNA expression profiles					
miRNAs downregulated in HCC with LNM	*P*-value	Fold changes	miRNAs upregulated in HCC with LNM	*P*-value	Fold changes
hsa-miR-129-3p	0.00167	35.467	hsa-miR-21	0.00344	28.892
hsa-miR-451	0.02672	31.102	hsa-miR-650	0.01723	27.294
hsa-miR-503	0.00321	30.008	hsa-miR-34a	0.00566	15.573
hsa-miR-26a	0.01305	29.776	hsa-miR-99b	5.12E-06	9.456
hsa-miR-214	3.78E-04	22.051	hsa-miR-494	0.00109	9.189
hsa-miR-491	0.00565	17.287	hsa-miR-135b	0.00502	8.425
hsa-miR-612	0.00376	12.515	hsa-miR-362-5p	0.00146	7.219
hsa-miR-106b	0.01008	10.084	hsa-miR-1228	0.02233	5.885
hsa-miR-9*	0.04571	9.433	hsa-miR-331-3p	0.00709	5.309
hsa-miR-337-5p	0.00175	8.105	hsa-miR-34a*	3.43E-04	5.122
hsa-miR-30c-2*	1.98E-05	6.677	hsa-miR-181c	0.02445	4.921
hsa-miR-145	0.00228	6.341	hsa-miR-193a-5p	4.18E-04	4.873
hsa-miR-377	0.03093	5.219	hsa-miR-16-2*	0.00553	4.721
hsa-miR-409-3p	0.00902	4.787	hsa-miR-106b	0.01078	3.968
hsa-miR-195	0.00279	3.654	hsa-miR-10b	0.02127	3.734
hsa-miR-153	0.00871	3.577	hsa-miR-155	0.00932	3.677
hsa-miR-218	0.01257	3.423	hsa-miR-17	0.00189	3.554
hsa-miR-660	0.00445	3.089	hsa-miR-30d	0.03144	3.407
hsa-miR-188-3p	0.04262	2.963	hsa-miR-28-3p	0.00232	3.264
hsa-miR-30c-2*	0.00774	2.814	hsa-miR-130b	1.79E-05	3.152
hsa-Let-7i	2.54E-04	2.672	hsa-miR-106a	0.02476	3.075
hsa-miR-520a-3p	0.01122	2.463	hsa-miR-204-5p	0.00664	2.911
hsa-miR-34a	0.00387	2.222	hsa-miR-181a	0.00117	2.872
hsa-miR-33b	0.02165	2.185	hsa-miR-143	0.02476	2.607
			hsa-miR-130b	0.00815	2.423
			hsa-miR-182	5.46E-06	2.409
			hsa-miR-374b	0.01992	2.312
			hsa-miR-615-3p	2.17E-04	2.272
			hsa-miR-149*	0.00405	2.187
			hsa-miR-182	0.00559	2.145
			has-miR-196a	0.02008	2.103
			hsa-miR-375	0.00613	2.068
			has-miR-20b	0.03537	2.059
			has-miR-127	0.00911	2.047

Note: Differentially expressed miRNAs were identified by using a filter based on a fold change of 2.0 combined with **P**<0.05 (ANOVA). HCC: hepatocelluar cancer; LNM: lymph node metastasis.

HCC tissues with LNM or vascular invasion showed lower miR-129-3p expression level than those tissues without LNM or vascular invasion (Figure 2B-2C). Also, low miR-129-3p was observed to correlate with advanced pTNM stage (Figure 2D). Most importantly, the relative expression level of miR-129-3p in tumors with recurrence was significantly lower than that in tumors without recurrence (Figure 2E). The correlations between miR-129-3p expression and clinicopathological characteristics were summarized in Supplementary Table 1, and statistically significant reverse associations between miR-129-3p expression and metastatic rates could be observed. By Kaplan-Meier survival analyses, it was found that reduced miR-129-3p was closely associated with shorter disease-free survival (DFS) and overall survival (OS) of HCC patients (Figure 2F). By univariate and multivariate analyses, low miR-129-3p expression was revealed to be an independent predictor for poorer DFS and OS in HCC patients (Supplementary Table 2-3).

Since miR-129-3p originates from miR-129-2, and the frequently hypermethylated miR-129-2 CpG island has been reported in other cancers [16, 17], we further hypothesized that such methylation would mediate miR-129-3p downregulation in HCC progression. The methylation level at the miR-129-2 promoter region was compared in HCC tissues and adjacent nontumor tissues by methylation-specific PCR, and a higher methylation level at the promoter region of miR-129-2 in HCC tissues could be observed (*P*<0.01; Supplementary Figure 1A). We further explored the correlation between expression level of miR-129-3p and methylation of miR-129-2 promoter, and showed that the expression level of miR-129-3p inversely correlated with methylation level of miR-129-2 promoter (*P*=0.019; Supplementary Figure 1B). Furthermore, HCC tissues with LNM or vascular invasion showed higher methylation level of miR-129-2 promoter than that those tissues without LNM (*P*<0.01) or vascular invasion (*P*<0.01) (Supplementary Figure 1C-1D). These data suggest that methylation-mediated miR-129-3p downregulation closely correlates with the increase of HCC metastatic potential.

### miR-129-3p significantly inhibits *in vitro* invasion and *in vivo* metastasis of HCC cells

To further determine the correlation between miR-129-3p expression and HCC metastasis, we detected miR-129-3p expression in HCC cell lines with different metastatic potentials, and showed that miR-129-3p decreased progressively from normal human hepatocyte cell line (HH) to low metastatic HCC cell lines (HepG2 and BEL-7402), and finally to highly metastatic HCC cell lines (HCCLM3 and MHCC97-H) (Supplementary Figure 2A). After treatment with a DNA methyltransferase inhibitor (10.0 μmol/L 5-Aza-dC), all of the 5-Aza-dC-treated HCC cells exhibited the significantly increased expression of miR-129-3p when compared with the control groups (Supplementary Figure 2B). To explore the biological significance of miR-129-3p in HCC cells, HCCLM3 or MHCC97-H cells were transiently transfected with or miR-NC/mimics or miR-129-3p/mimics and HepG2 or BEL-7402 cells were transiently transfected with anti-miR-NC or anti-miR-129-3p, respectively. First, qRT-PCR confirmed the upregulation or downregulation of miR-129-3p in those transfected cells (Supplementary Figure 3A-4A). Re-expression of miR-129-3p in HCCLM3 or MHCC97-H cells induced moderate suppression of *in vitro* cell growth and *in vivo* tumorigenesis, and G_0_/G_1_ cell cycle arrest, but no obvious apoptosis (Supplementary Figure 3B-3F). In contrast, miR-129-3p downregulation in HepG2 and BEL-7402 cells increased cell growth, but induced no obvious changes in cell cycle and apoptosis (Supplementary Figure 4B-4E).

**Figure 3:**
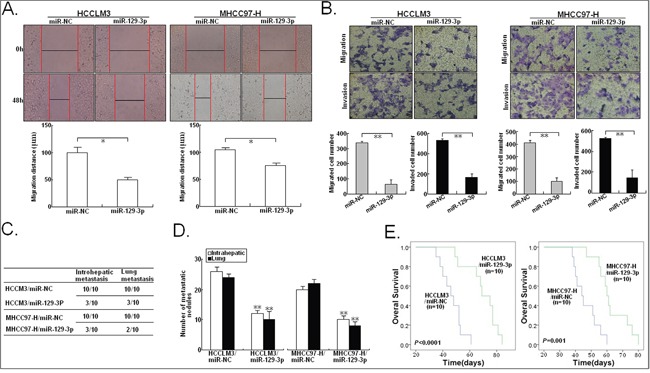
miR-129-3p inhibits *in vitro* migration or invasion and *in vivo* metastasis in HCC cells **A.** Wound healing assay. A confluent monolayer of miR-NC/mimics or miR-129-3p/mimics-transfected HCCLM3 or MHCC97-H cells was wounded. Photographs were taken immediately (0 h) and at 48 h after wounding, quantification of wound closure was done. The data present the mean distance of cell migration to the wound area at 48 h after wounding in three independent wound sites per group. **B.** Transwell migration and invasion assay of miR-NC/mimics or miR-129-3p/mimics-transfected HCCLM3 or MHCC97-H cells. Cells in six random fields of view at 100× magnification were counted and expressed as the average number of cells per field of view. **C.** Incidence of intrahepatic or lung metastasis in different groups of nude mice transplanted with miR-NC/mimics or miR-129-3p/mimics-transfected HCCLM3 or MHCC97-H cells (n=10/group). **D.** Hematoxylin and eosin staining of intrahepatic and lung metastatic tumor nodules formed from miR-NC/mimics or miR-129-3p/mimics-transfected HCCLM3 or MHCC97-H cells (n=10/group). The numbers of metastatic nodules in each nude mice were counted and statistically analyzed. **E.** The OS time of different groups of nude mice transplanted with miR-NC/mimics or miR-129-3p/mimics-transfected HCCLM3 or MHCC97-H cells (n=10/group). The survival data were compared with the log-rank test. ^*^P<0.05; ^**^P<0.01.

Further, we investigated the roles of miR-129-3p in migration and invasion of HCC cells. Wound healing assays showed that miR-129-3p upregulation significantly reduced the capacity of mobility in HCCLM3 and MHCC97-H cells (Figure 3A). In contrast, miR-129-3p downregulation increased wound healing of HepG2 and BEL-7402 cells (Supplementary Figure 5A). Similarly, in Transwell assays, miR-129-3p upregulation markedly inhibited migration and invasion of HCCLM3 and MHCC97-H cells (Figure 3B). Conversely, miR-129-3p downregulation increased migration and invasion of HepG2 and BEL-7402 cells (Supplementary Figure 5B). Then, to investigat whether miR-129-3p upregulation affects the *in vivo* metastasis of HCC cells, HCCLM3/miR-129-3p or MHCC97-H/miR-129-3p cells were transplanted into the left hepatic lobe of nude mice. By histological analysis, it was observed that the incidence and number of both intrahepatic and lung metastasis in the HCCLM3/miR-129-3p or MHCC97-H/miR-129-3p group was significantly decreased, in compared with the control group (Figure 3C-3D). The HCCLM3/miR-129-3p or MHCC97-H/miR-129-3p group lived longer than the control group (Figure 3E). Likewise, the effect of miR-129-3p downregulation on the *in vivo* metastasis of HCC cells was also determined. It was observed that the number of both intrahepatic and lung metastasis in the HepG2/anti-miR-129-3p or BEL-7402/anti-miR-129-3p group was significantly decreased, as compared to the control group (Supplementary Figure 5C). The HepG2/anti-miR-129-3p or BEL-7402/anti-miR-129-3p group lived shorter than the control group (Supplementary Figure 5D). These results demonstrated that miR-129-3p reduces invasion and metastasis of HCC cells.

### miR-129-3p reverses EMT in HCC cells

**Figure 4:**
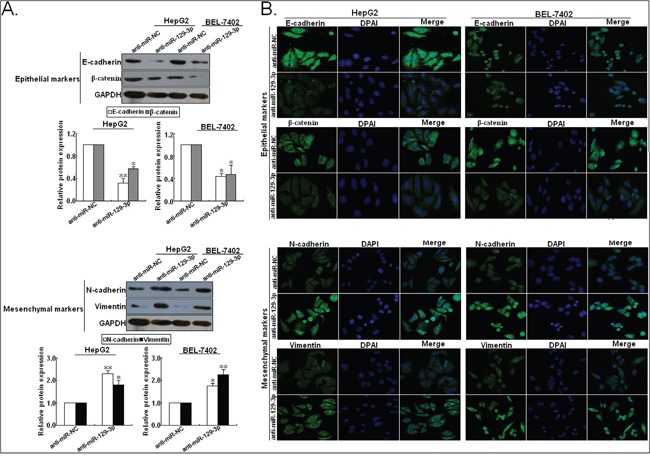
Upregulation of miR-129-3p reverses EMT in HCC cells (high initial metastatic potential) **A.** Western blotting and **B.** immunofluorescence staining assays indicated that the increased expression of epithelial markers (E-cadherin and β-catenin) and the decreased expression of mesenchymal markers (N-cadherin and Vimentin) could be obviously observed in miR-129-3p/mimics-transfected HCCLM3 or MHCC97-H cells, compared with miR-NC/mimics-transfected cells. Each experiment was performed in triplicate.^*^P<0.05; ^**^P<0.01.

EMT has been postulated as an absolute requirement for tumor invasion and metastasis [18]. Immunofluorescence and Western blotting assays indicated that miR-129-3p upregulation significantly increased the expression of epithelial markers (E-cadherin and β-catenin) and decreased the expression of mesenchymal markers (N-cadherin and Vimentin) in HCCLM3 and MHCC97-H cells (Figure 4A-4B). In contrast, miR-129-3p downregulation induced the decreased expression of epithelial markers and increased expression of mesenchymal markers in HepG2 and BEL-7402 cells (Supplementary Figure 6A-6B). Thus, upregulation of miR-129-3p reverses EMT phenotype of HCC cells.

### Identification of Aurora-A as a direct target of miR-129-3p

**Figure 5:**
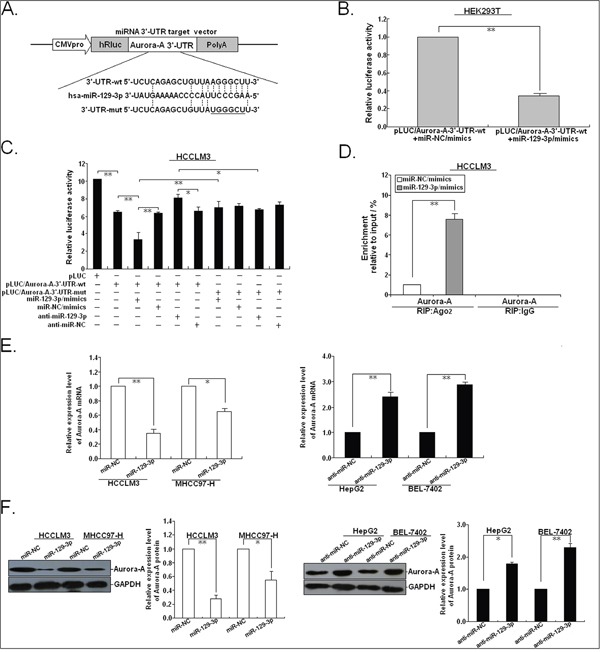
MiR-129-3p binds to the 3′-UTR of human Aurora-A mRNA **A.** Mutation was generated on the Aurora-A 3′-UTR sequence in the complementary site for the seed region of miR-129-3p, as shown. A human Aurora-A 3′-UTR fragment containing wild-type or mutant miR-129-3p-binding sequence was cloned into downstream of the luciferase reporter gene in pLUC-luc vector. **B.** pLUC vector contains Aurora-A mRNA 3′-UTR and miR-129-3p/mimics or control miR-NC/miR-mimics were co-transfected into HEK293T cells, Cells lysates were prepared after 48 h for measuring luciferase activity, which was normalized to normalized to Renilla luciferase activity. **C.** Relative luciferase activity was analyzed after wildtype or mutant 3′-UTR reporter plasmids were co-transfected with miR-129-3p/mimics or anti-miR-129-3p in HCCLM3 cells. The histogram shows the mean±SEM of the normalized luciferase activity from three independent experiments. **D.** The RIP analysis revealed recruitment of Aurora-A mRNAs to miRNP complex in miR-129-3p/mimics (or miRNA-NC/mimics)-transfected HCCLM3 cells following immunoprecipitation against Ago2. The IgG immunoprecipitation was used as a negative control. **E.** qRT-PCR detection of Aurora-A mRNA expression in miR-129-3p/mimics (or miR-NC/mimics)-transfected HCCLM3 or MHCC97-H cells and anti-miR-129-3p (or anti-miR-NC)-transfected HepG2 or BEL-7402 cells. **F.** Western blotting detection of Aurora-A protein expression in miR-129-3p/mimics (or miR-NC/mimics)-transfected HCCLM3 or MHCC97-H cells and anti-miR-129-3p (or anti-miR-NC)-transfected HepG2 or BEL-7402 cells. GAPDH was used as an internal control. ^*^P<0.05; ^**^P<0.01.

To dissect the possible molecular mechanisms by which miR-129-3p functions in HCC cells, we searched for candidate target genes regulated by miR-129-3p using a dual-pronged approach of in silico target prediction using two open access software (miRanda and TargetScan), and found that Aurora-A might be a putative target of miR-129-3p. Aurora-A, a serine/threonine protein kinase, is essential for mitosis and has a crucial role in tumorigenesis. In particular, in HCC, our and other studies have shown that overexpression of Aurora-A correlates with HCC development and poor patient prognosis [19, 20]. For this reason, this gene was investigated further. In silico analysis indicated that 3′-UTR of human Aurora-A (2247-22672nt) involves a potential miR-129-3p binding site. To testify this possibility, the miR-129-3p binding sequences present at the 3′-UTR of Aurora-A mRNA (3′-UTR-wt) or its mutant (3′-UTR-mut) were subcloned into the downstream of the firely luciferase reporter gene in pLUC vector, which named pLUC/Aurora-A-3′-UTR-wt and pLUC/Aurora-A-3′-UTR-mut, respectively (Figure 5A). First, pLUC/Aurora-A-3′-UTR-wt and pLMP/miR-129-3p or pLMP-miR-129-3p were co-transfected into human HEK 293T cells. At 48h after co-transfection, the luciferase activity was determined. Results showed that the luciferase activity was significantly suppressed by 65.4% in HEK293T cells co-transfected with pLUC/Aurora-A-3′-UTR-wt and pLMP/miR-129-3p (*P*<0.01), when compared with the control groups (Figure 5B). To further determine whether miR-129-3p binds to the 3′-UTR region of Aurora-A mRNA, pLUC/Aurora-A-3′-UTR-wt (or pLUC/Aurora-A-3′-UTR-mut) and pLMP/miR-129-3p (or pLMP/miR-NC) or anti-miR-129-3p (or anti-miR-NC) were co-transfected into HCCLM3 cells, and luciferase activity was determined (Figure 5C). The luciferase activity was reduced by almost 47.7% by pLMP/miR-129-3p (*P*<0.01) when the wildtype 3′-UTR of Aurora-A was present, and the activity was increased by 27.2% (*P*<0.05) when miR-129-3p was downregulated. However, the mutated 3′-UTR of Aurora-A prevented miR-129-3p expression from affecting the luciferase activity. In addition, RNA-immunoprecipitation (RIP) analysis revealed that miR-129-3p upregulation enhanced the recruitment of Aurora-A mRNA to miRNP complexes (Figure 5D). qRT-PCR (Figure 5E) and Western blotting (Figure 5F) assays were performed in HCC cells stably transfected with pLMP/miR-129-3p (or pLMP/miR-NC) or transiently transfected with anti-miR-129-3p (or anti-miR-NC). The expression levels of Aurora-A mRNA and protein in HCCLM3 and MHCC97-H cells stably transfected with pLMP/miR-129-3p were observed to be significantly downregulated, when compared with pLMP/miR-NC-transfected cell. Meanwhile, at 48h after transient transfection, the expression levels of Aurora-A mRNA and protein in both cells transfected with anti-miR-129-3p were significantly upregulated, when compared with anti-miR-NC-transfected cells. These results suggested that miR-129-3p negatively regulates Aurora-A by directly binding to the 3′-UTR of Aurora-A mRNA in HCC cells.

### Suppression of Aurora-A mimics the biological functions of miR-129-3p in HCC cells

**Figure 6:**
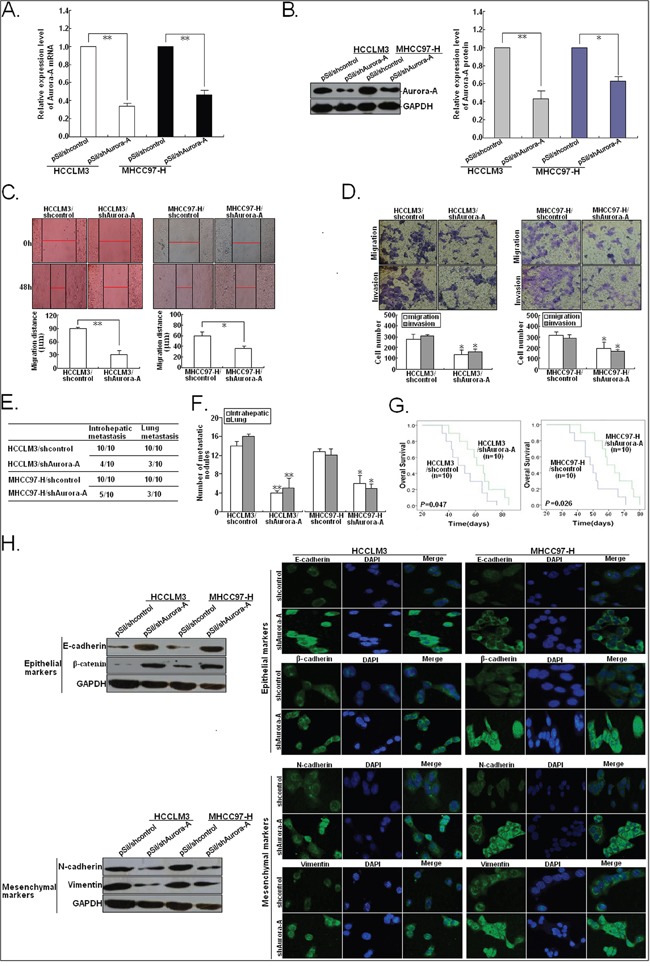
Silencing of Aurora-A inhibits *in vitro* invasion and *in vivo* metastatic capacity, and reverses EMT in HCC cells **A.** qRT-PCR detection of Aurora-A mRNA expression in pSil/shcontrol or pSil/shAurora-A-transfected HCCLM3 or MHCC97-H cells. **B.** Western blotting detection of Aurora-A protein expression in pSil/shcontrol or pSil/shAurora-A-transfected HCCLM3 or MHCC97-H cells. **C.** Wound healing assay. A confluent monolayer of pSil/shcontrol or pSil/shAurora-A-transfected HCCLM3 or MHCC97-H cells was wounded. **D.** Transwell migration and invasion assay of pSil/shcontrol or pSil/shAurora-A-transfected HCCLM3 or MHCC97-H cells. **E.** Incidence of intrahepatic or lung metastasis in different groups of nude mice transplanted with pSil/shcontrol or pSil/shAurora-A-transfected HCCLM3 or MHCC97-H cells (n=10/group). **F.** Hematoxylin and eosin staining of intrahepatic and lung metastatic tumor nodules formed from pSil/shcontrol or pSil/shAurora-A-transfected HCCLM3 or MHCC97-H cells (n=10/group). The numbers of metastatic nodules in each nude mice were counted and analyzed using Student *t* test. **G.** The OS time of different groups of nude mice transplanted with pSil/shcontrol or pSil/shAurora-A-transfected HCCLM3 or MHCC97-H cells (n=10/group). **H.** Western blotting and immunofluorescence staining detection of epithelial markers and mesenchymal markersin pSil/shcontrol or pSil/shAurora-A-transfected HCCLM3 or MHCC97-H cells. Each experiment was performed in triplicate. ^*^P<0.05; ^**^P<0.01.

To investigate whether Aurora-A is a functional target gene of miR-129-3p in HCC cells, we first determined whether small hairpin RNA (shRNA)-mediated Aurora-A kncoddown mimics the effects of miR-129-3p upregulation. The results of qRT-PCR and Western blotting confirmed the knockdown of Aurora-A in HCCLM3/shAurora-A or MHCC97-H/shAurora-A cells (Figure 6A-6B). As expected, the *in vitro* mobility, migration and invasion of HCCLM3/shAurora-A and MHCC97-H/shAurora-A cells could be significantly inhibited, in comparison with the control group (Figure 6C-6D). In addition, the effects of Aurora-A knockdown on the *in vivo* metastasis of HCC cells were also analyzed. By histological analysis, we showed that all of the cases in the control group formed intrahepatic or lung metastasis. However, only 4 (or 5) cases of intrahepatic metastasis and 3 (or 3) cases of lung metastasis could be observed in HCCLM3/shAurotra-A or MHCC97-H/shAurora-A group (Figure 6E). Also, the number of intrahepatic or lung metastatic nodules in the HCCLM3/sure 6F). The HCCLM3/shAurora-A or MHCC97-H/shAurora-A group lived longer than the control group (Figure 6G). Meanwhile, knockdown of Aurora-A significantly increased epithelial markers and decreased mesenchymal markers in HCCLM3 and MHCC97-H cells (Figure 6H). Thus, knockdown of Aurora-A mimics the effects of miR-129-3p upregulation on phenotypes of HCC cells.

### Restoration of Aurora-A partially rescues miR-129-3p-mediated EMT, invasion and metastasis in HCC cells

**Figure 7:**
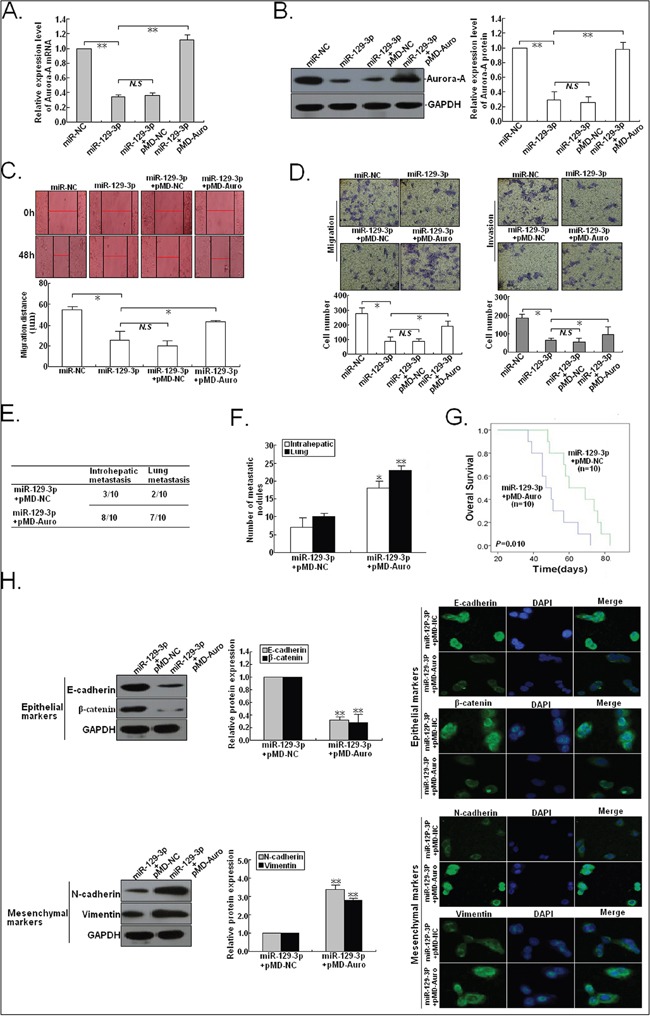
Overexpression of Aurora-A reverses the effects of miR-129-3p upregulation on HCC cells 48h after HCCLM3 cells were co-transfected with miR-129-3p/mimics and pMD-Auro (or pMD-NC), **A.** qRT-PCR and **B.** Western blotting detection of Aurora-A mRNA and protein expression. GAPDH was used as an internal control. **C.** Wound healing assay. A confluent monolayer of HCCLM3 cells co-transfected with miR-129-3p/mimics and pMD-Auro (or pMD-NC) was wounded. **D.** Transwell migration and invasion assay of HCCLM3 cells co-transfected with miR-129-3p/mimics and pMD-Auro (or pMD-NC). **E.** Incidence of intrahepatic or lung metastasis in different groups of nude mice transplanted with HCCLM3 cells co-transfected with miR-129-3p/mimics and pMD-Auro (or pMD-NC) (n=10/group). **F.** Hematoxylin and eosin staining of intrahepatic and lung metastatic tumor nodules formed from HCCLM3 cells co-transfected with miR-129-3p/mimics and pMD-Auro (or pMD-NC) (n=10/group). The numbers of metastatic nodules in each nude mice were counted and analyzed. **G.** The OS time of different groups of nude mice transplanted with HCCLM3 cells co-transfected with miR-129-3p/mimics and pMD-Auro (or pMD-NC) (n=10/group). The survival data were compared with the log-rank test. **H.** Western blotting and immunofluorescence staining detection of expression of epithelial markers (E-cadherin and β-catenin) and mesenchymal markers (N-cadherin and Vimentin) in HCCLM3 cells co-transfected with miR-129-3p/mimics and pMD-Auro or pMD-NC. Each experiment was performed in triplicate. ^*^P<0.05; ^**^P<0.01.

Then, we rescued expression of Aurora-A in HCCLM3/miR-129-3p cells by transfecting the plasmid vector carrying WT Aurora-A (pMD-Auro) into HCCLM3/miR-129-3p cells. Western blotting showed that transfection of pMD-Auro rescued the decreased expression of Aurora-A protein in HCCLM3/miR-129-3p cells (Figure 7A-7B). The migratory and invasive capacity of HCCLM3/miR-129-3p cells was also partially rescued after transfection of pMD-Auro (Figure 7C-7D). By *in vivo* metastatic assays, it was confirmed that 8 mice developed intrahepatic metastasis and 7 mice developed lung metastasis in the HCCLM3/miR-129-3p/pMD-Auro group. However, only 3 cases of intrahepatic metastasis and 2 cases of lung metastasis in the control group could be observed (Figure 7E). Also, the increased number of intrahepatic or lung metastatic nodules in the HCCLM3/miR-129-3p/pMD-Auro group could be observed, when compared with the control group (Figure 7F). Compared to the contro group, the HCCLM3/miR-129-3p/pMD-Auro group showed a shorter OS time (Figure 7G). Meanwhile, we also found that re-expression of Aurora-A could induce EMT in HCCLM3/miR-129-3p cells by downregulating epithelial markers and upregulating mesenchymal markers (Figure 7H). Thus, re-expression of Aurora-A partially rescues miR-129-3p-mediated EMT, invasion and metastasis in HCC cells.

### The PI3K/Akt and p38-MAPK signalings are involved in miR-129-3p/Aurora-A-mediated effects on phenotypes in HCC cells

**Figure 8:**
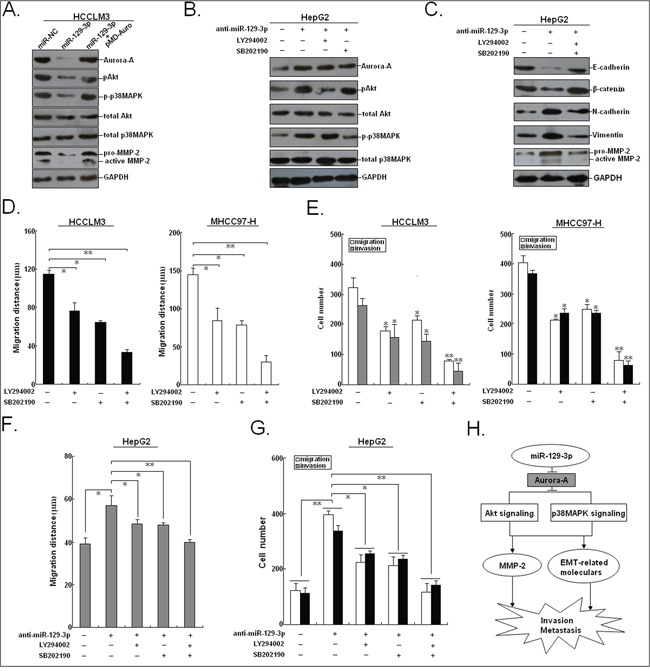
miR-129-3p and Aurora-A are involved in PI3K/Akt and p38-MAPK signalings in HCC cells **A.** Western blotting detection of Aurora-A, pAkt, p-p38-MAPK, total Akt, total p38-MAPK and MMP-2 proteins in miR-NC/mimics or miR-129-3p/mimics-transfected HCCLM3 cells or HCCLM3 cells co-transfected with miR-129-3p/mimics and pMD-Auro. **B.** Western blotting detection of Aurora-A, p-Akt, p-p38-MAPK, total Akt, total p38-MAPK proteins in anti-miR-NC or anti-miR-129-3p-transfected HepG2 cells, followed by the treatment of the inhibitor of p38-MAPK (SB202190) or Akt (LY294002). **C.** Western blotting detection of EMT-related proteins in anti-miR-NC or anti-miR-129-3p-transfected HepG2 cells, followed by the treatment of SB202190 or LY294002. **D.** Wound healing assay. A confluent monolayer of HCCLM3 or MHCC97-H cells treated with SB202190 or LY294002 was wounded. The data present the mean distance of cell migration to the wound area at 48 h after wounding in three independent wound sites per group. **E.** Transwell migration and invasion assay of HCCLM3 or MHCC97-H cells treated with SB202190 or LY294002. **F.** Wound healing and **G.** transwell migration and invasion assays of HepG2 cells transfected with anti-miR-NC or anti-miR-129-3p, followed by the treatment of SB202190 or LY294002. **H.** Schematic overview of miR-129-3p regulatory signaling. GAPDH was used as an internal control. ^*^P<0.05; ^**^P<0.01.

It has been reported that the PI3K/Akt and p38-MAPK signalings were involved in Aurora-A-induced tumor EMT, invasion as well as MMP-2 activity in a variety of human cancers [21, 22]. Therefore, we investigated whether miR-129-3p suppressed invasion and metastasis of HCC cells via effects the p38-MAPK and PI3K/Akt signalings by targeting Aurora-A. The expression levels of phosphorylated Akt (p-Akt) and p38-MAPK (p-p38-MAPK) were significantly downregulated in HCCLM3/miR-129-3p cells compared to HCCLM3/miR-NC cells, with downregulation of MMP-2 (Figure 8A). However, ectopic Aurora-A expression could rescue the downregulated expression of p-Akt, p-p38-MAPK and MMP-2 proteins in HCCLM3/miR-129-3p cells. Furthermore, HepG2 cells transfected with anti-miR-129-3p or anti-miR-NC were treated with a selective inhibitor of p38-MAPK (SB202190) or Akt (LY294002) for 48h, respectively. The results (Figure 8B) indicated that SB202190 suppressed activation of p-p38MAPK, but not p-Akt, whereas LY294002 treatment induce opposite effects, suggesting that SB202190 or LY294002 could reverse activation of p38-MAPK and Akt signalings induced by anti-miR-129-3p. Moreover, we found that combined treatment (SB202190 and LY294002) could reverse the decreased epithelial markers and the increased mesenchymal markers or MMP-2 protein in anti-miR-129-3p-transfected HepG2 cells (Figure 8C). We further showed that SB202190 or LY294002 treatment significantly reduced migration and invasion in HCCLM3 and MHCC97-H cells, and the combination treatment exerted a stronger inhibitory effect (Figure 8D-8E). Also, treatment of SB202190 or/and LY294002 partially rescues the anti-miR-129-3p-promoting effects on migration and invasion of HCC cells (Figure 8F-8G). Therefore, it is concluded that miR-129-3p inhibits HCC migration and invasion by inactivation of PI3K/Akt and p38-MAPK signalings, which eventually induces the reversal of EMT phenotype and downregulation of MMP-2 in HCC cells (Figure 8H).

### Aurora-A is upregulated in HCC tissues and inversely correlates with miR-129-3p expression

**Figure 9:**
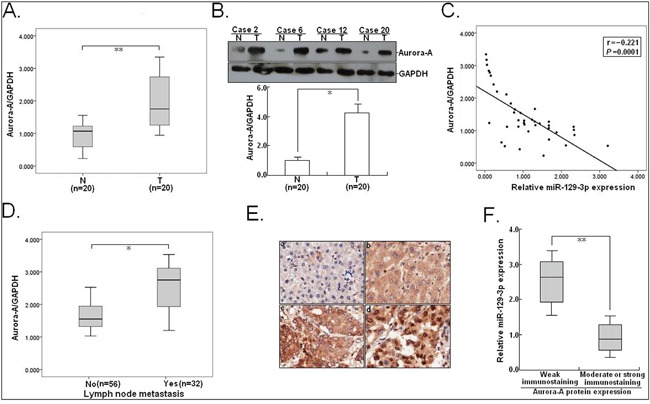
Aurora-A is significantly upregulated in HCC tissues and negatively correlates with miR-129-3p **A.** qRT-PCR detection of Aurora-A mRNA expression in HCC tissues and corresponding nontumor liver tissues (n=20). T: HCC tissues; N: nontumor liver tissues. **B.** Western blotting detection of Aurora-A protein expression in HCC tissues and corresponding nontumor liver tissues (n=20). **C.** Statistically significant inverse correlation between miR-129-3p and Aurora-A protein expression in 20 cases of HCC tissues (r = −0.221; *P*=0.0001). **D.** qRT-PCR detection of Aurora-A mRNA expression in HCC tissues without LNM (n=56) and HCC tissues with LNM (n=32). **E.** Immunostaining of Aurora-A protein was negatively or very weakly positive in corresponding nontumor liver tissues (a and b), but was moderately or strongly positive in HCC tissues (c and d). Origninal magnification, ×100. **F.** The immunoreactivity of Aurora-A protein in HCC tissues showed a statistically significant inverse correlation the relative level of miR-129-3p expression (*P*<0.01). Each experiment was performed in triplicate. GAPDH was used as an internal control. Data were presented as mean±SEM (n=3). ^*^P<0.05; ^**^P<0.01.

To address the biological significance of the miR-129-3p/Aurora-A interaction in HCC, qRT-PCR and Western blotting were done to detect expression of Aurora-A mRNA and protein in HCC tissues and corresponding nontumor tissues (n=20). It was confirmed that HCC tissues showed higher Aurora-A mRNA and protein expression levels than the corresponding nontumor tissues (Figure 9A-9B). By linear regression analysis, a significant inverse association between miR-129-3p and Aurora-A mRNA expression could be observed (r = −0.221; *P* = 0.0001; Figure 9C). Further, the expression level of Aurora-A mRNA in tumor tissues with LNM was higher than tumor tissues without LNM (*P*<0.05; Figure 9D). Next, immunohistochemistry was performed to detect Aurora-A protein expression in 88 HCC tissues (Figure 9E). According to the immunoreactive intensity of Aurora-A protein, 46 patients were classified as high-Aurora-A group (3+ or 2+) and 42 as low-Aurora-A group (1+ or 0). The stronger immunoreactivity of Aurora-A in HCC tissues was observed to be significantly correlated with lower miR-129-3p expression (*P*<0.001; Figure 9F). Therefore, upregulation of Aurora-A closely correlates with downregulated miR-129-3p in HCC tissues.

## DISCUSSION

In the present study, we showed that promoter methylation of miR-129-3p could lead to miR-129-3p downregulation in HCC, and upregulation of miR-129-3p suppressed HCC cell EMT, *in vitro* invasion and *in vivo* metastasis through post-transcriptionally regulating Aurora-A. The data from the current study suggest that miR-129-3p plays a crucial role in liver carcinogenesis, especially in the process of HCC metastasis.

Besides protein-encoding genes, miRNAs have more than a cursory role during the pathogenesis of tumor progression, including metastasis [23]. To date, abberant miRNAs have been reported to play important roles in HCC development, and some of them have been identified to be prognostic biomarkers or potential therapeutic targets [24, 25]. However, elucidation of miRNA deregulation or dysfunction in HCC metastasis is still underway. We compared the miRNA expression patterns between HCC with and without LNM using miRNAs microarray, and found that 11 miRNAs correlates with LNM of HCC. Among them, miR-129-3p was one of the most downregulated miRNA. Recently, two members of the hsa-miR-129 family have been identified: hsa-miR-129-1 and 2. Meanwhile, miR-129-5p and 3p are originated from miR-129-2. Recent studies have shown that dysregulation of miR-129 family members is a frequent event in many human cancers. For example, epigenetic repression of microRNA-129-2 has been reported to induce overexpression of SOX4 in endometrial cancer, gastric cancer and HCC [16, 17, 26]. Additionally, Yu’ et al showed that three miR-129 family members play an important role in regulating gastric cancer cell proliferation [27]. Karaayvaz’ et al showed that miR-129 promotes apoptosis and enhances 5-fluorouracil chemosensitivity in colorectal cancer [28]. In HCC, miR-129-5p was reported to inhibit HCC metastasis by targeting ETS1 [29]. Most importantly, miR-129-5p was found to reduce the degradation of IκBα and increase apoptosis and reduce migration of HCC cells by downregulating VCP/p97 [30]. However, the roles of miR-129-3p in HCC metastasis and its possible mechanisms remain to be further elucidated.

Here, miR-129-3p was downregulated in HCC tissues, and patients with low miR-129-3p tended to have more advanced TNM stage and higher incidence of LNM, vascular invasion and tumor recurrence. Moreover, HCC patients with high miR-129-3p had better prognosis than those with low miR-129-3p, and multivariate analysis indicated that expression of miR-129-3p might be an independent factor for the prediction of recurrence and survival in HCC patients. DNA methylation has emerged as one of the mechanisms in the transcriptional silencing of tumor suppressor genes or suppressor miRNAs during tumor development [31]. Recently, the miR-129-2 epigenetic silencing by methylation is reported to be a frequent event in human cancers. In HCC, Lu et al showed that status of miR-129-2 methylation is highly accurate in distinguishing HCC patients from cirrhosis patients and healthy individuals [32]. Also, Liu et al reported that DNA methylation-mediated inhibition of microRNA-129-2 reduces HCC cell aggressiveness by downregulating high mobility group box 1 [33]. So far, the respective roles of three miR-129-2 family members (miR-129-3p or 5p) in HCC development are not fully understood. Therefore, the aim of our study is to determine the roles of methylation-mediated miR-129-3p downregulation in HCC development, especially metastasis. We first determined the methylation status of miR-129-2 in HCC tissues using methylation-specific PCR and HCC cells with or without 5-Aza-dC treatment, and showed that promoter methylation level of miR-129-2 was higher in HCC tissues compared to adjacent nontumor tissues and inversely correlated with miR-129-3p expression. Those results were further confirmed in another study from Chen et al [26]. We also found that the miR-129-2 CpG methylation level was higher in HCC tissues with LNM or vascular invasion compared to tissues without LNM or vascular invasion. In addition, we showed that 5-Aza-dC treatment restored miR-129-3p expression in HCC cells. These data clearly suggest that promoter methylation-mediated miR-129-3p downregulation may play critical roles in HCC progression. Next, gain - and loss - of - function assays were performed to investigate the roles of miR-129-3p expression in HCC metastasis. Indeed, our data demonstrate that miR-129-3p upregulation significantly reduced migration and invasion of highly metastatic HCC cells, while miR-129-3p downregulation could enhance migration and invasion of low metastatic HCC cells. Xenograft tumor assays provided additional support for involvement of miR-129-3p in suppressing hepatic and lung metastasis *in vivo*. EMT plays important roles in HCC metastasis, and the associations of miRNAs with HCC EMT are also increasingly reported [34]. Here we showed that miR-129-3p upregulation exerted obvious effects on EMT phenotypes of HCC cells by increasing epithelial markers and decreasing mesenchymal markers. In contrast, miR-129-3p downregulation could induce opposite changes of those markers. As EMT is thought to be a key step for tumor metastasis, HCC cells with low miR-129-3p expression become more invasive. These data demonstrated that miR-129-3p functions as a metastasis suppressor in HCC cells through reversing EMT and suppressing invasion and metastasis.

To further elucidate the underlying mechanisms by which miR-129-3p exerts its functions, we need to determine its direct targets by using a dual-pronged approach of in silico target prediction using two algorithms. According to the functions of those gene and the effects of miR-129-3p on metastasis of HCC cells, Aurora-A was chosen as the interesting target gene in our further studies. Aurora-A belongs to Aurora kinase family, and is reported to be overexpressed in various human cancers, including HCC [35]. Our previous studies have shown that high Aurora-A expression significantly correlates with frequent lymph node metastasis of HCC patients, and silencing of Aurora-A inhibits growth and enhances apoptosis in HCC cells [19, 36]. However, the mechanisms underlying Aurora-A overexpression in HCC are fully unclear. Transcriptional control and post-transcriptional control are critical regulatory mechanisms for gene expression. Previously, we have reported that hypoxia could activate Aurora A transcription by increasing the recruitment of HIF-1α to potential hypoxia-responsive elements on its promoter [37]. Recently, miRNAs are now recognized as key post-transcriptional regulators of gene expression. Although miR-199a-3p has been reported to attenuate xenograft tumor growth of prostate carcinoma by targeting Aurora-A [38], the expression of miR-199a-3p showed no difference between HCC tissues with or without LNM from our miRNA array data and by qRT-PCR (data not shown). A single gene can be regulated by several miRNAs, while a single miRNA can regulate multiple target genes within the same or different tissues. Thus, it is needed to identify other miRNAs which target Aurora-A to play critical roles in HCC metastasis. Our data clearly suggested that miR-129-3p inhibited invasion and metastasis of HCC cells by targeting Aurora-A. This conclusion was based on several experimental data. First, miR-129-3p upregulation significantly downregulated Aurora-A expression in HCC cells, while miR-129-3p downregulation significantly upregulated Aurora-A expression. Second, the luciferase assay with a reporter containing the wild miR-129-3p binding sequence at the 3′-UTR of Aurora-A mRNA indicated that the luciferase activity could be significantly reduced by miR-129-3p. Third, knockdown of Aurora-A could mimic the effects of miR-129-3p upregulation, whereas upregulation of Aurora-A could partially reverse the effects of miR-129-3p upregulation. Fourth, expression of Aurora-A in HCC tissues was observed to negatively correlate with miR-129-3p expression. Thus, these results revealed that dysregulation of miR-129-3p/Aurora-A might play critical roles in HCC metastasis.

To further better define the functions of miR-129-3p in HCC metastasis, we further explored the possible signalings which were involved in the regulation of miR-129-3p. The PI3K/Akt and p38-MAPK signalings have important implications in the regulation of tumor EMT and metastasis [39, 40]. Our and other researches have shown that overexpression of Aurora-A can activate those two signalings in human cancers. For example, Wan’ et al showed that inhibition of Aurora-A suppresses EMT by downregulating MAPK in nasopharyngeal carcinoma cells [21]. Also, overexpression of Aurora-A promotes ESCC development by enhancing invasion as well as MMP-2 expression in tumor cells, which can occur by activating p38-MAPK and Akt signalings [22]. Based on above experimental results, it can be hypothesized that miR-129-3p may reverse EMT and inhibit metastasis in HCC cells via effects on the PI3K/Akt and p38-MAPK signalings by targeting Aurora-A. Specifically, we found that both miR-129-3p upregulation and Aurora-A downregulation induced the decreased expression of phosphorylated Akt and p38-MAPK proteins, which followed by the increased epithelial markers and the decreased mesenchymal markers or MMP-2. MMPs, a large family of calcium-dependent zinc-containing endopeptidases, are responsible for the tissue remodeling and degradation of the extracellular matrix, and high levels of certain MMPs are associated with tumor metastasis [41, 42]. Thus, downregulation of MMP-2 is also one important mechanism by which miR-129-3p suppresses HCC metastasis. We found that upregulation of Aurora-A partially restored the changes in the expression of those proteins induced by miR-129-3p upregulation. Furthermore, the p38-MAPK or Akt inhibitors could reverse not only the increased aggressive capacities in HCC cells but also the increased expression of p-Akt and p-p38-MAPK proteins induced by miR-129-3p downregulation. Therefore, miR-129-3p and Aurora-A regulate HCC cell migration and invasion by mediating PI3K/Akt and p38-MAPK signalings.

Taken together, our study indicated that miR-129-3p was silencing in HCC through promoter hypermethylation and reduced miR-129-3p correlates with tumor metastasis and poor survival in HCC. Re-expression of miR-129-3p reverses EMT, inhibits *in vitro* invasion and *in vivo* metastasis of HCC cells by inactivating PI3K/Akt and p38-MAPK signalings which are partially through targeting Aurora-A. Therefore, miR-129-3p may be a promising prognostic biomarker and represent a potential therapeutic target in HCC.

## MATERIALS AND METHODS

### Patients and tissue samples

During the period from 2005 to 2007, primary HCC and corresponding nontumor liver tissues were obtained from 88 patients who received surgery at the Liver Disease Center of the 81th Hospital of PLA and the Department of Hepatobiliary Surgery of First Hospital Affiliated to the Chinese PLA General. Hospital. All tumors were histopathologically confirmed to contain at least 80% malignant cells and none of the participants received preoperative treatment. The tumor type and the grade of cell differentiation were designated based on the criteria of World Health Organization (WHO), whereas the pathological stage of each tumor was determined by the International Union Against Cancer (UICC) TNM classification. Details of clinical characteristics of the patients were shown in Supplementary Table 1. All patients were under a close follow-up observation for disease recurrence at 1-month intervals during the first 2 postoperative years, and every 3 months thereafter. Except tissues used for RNA extraction, the remnant tissues were rapidly frozen in liquid nitrogen and stored at -80°C. Ethical approval was obtained from the hospital and fully informed consent from all patients prior to sample collection. This study was approved by the Review Board of Hospital Ethics Committee (Jingling Hospital, Nanjing University).

### Cell lines and mice

Four HCC cell lines (HCCLM3, MHCC97-H, HepG2 and BEL-7402) were cultured in RPMI 1640 (GIBCO-BRL) medium supplemented with 10% fetal bovine serum (FBS), 100 U/ml penicillin, and 100 μg/ml streptomycin in humidified air at 37°C with 5% CO_2_. A normal human hepatocyte cell line (HH) and an immortalized human embryonic kidney cell line 293T (HEK293T) were cultured in Dulbecco's modified Eagle's medium (DMEM, GIBCO-BRL) supplemented with 10% fetal bovine serum (FBS), 100 U/ml penicillin, and 100 μg/ml streptomycin. All cells were fostered in a humidified atmosphere of 5% CO_2_ and 95% air. All female BALB/c athymic nude mice at 5 to 6 weeks of age in this study were purchased from the Department of comparative medicine, Jingling Hospital (School of Medicine, Nanjing University) and housed in laminar flow cabinets under specific pathogen-free conditions with food and water provided adlibitum. All animal experiments were performed with the approval of the Institutional Committee for Animal Research and in conformity with national guidelines for the care and use of laboratory animals. The study protocol was also approved by the Committee on the Use of Live Animals in research, Nanjing University (Nanjing, China).

### RNA extraction and microRNA microarray assay

For microRNA microarray assay, 4 cases of HCC tissues with LNM (HCC-L1, HCC-L2, HCC-L3 and HCC-L4) and 4 cases of HCC without LNM (HCC-LN1, HCC-LN2, HCC-LN3 and HCC-LN4) were collected from HCC patients who received surgery at the Liver Disease Center of the 81th Hospital of PLA. All tumors were histopathologically confirmed to contain at least 80% malignant cells and none of the participants received preoperative treatment. The characteristics of patients were shown in Supplementary Table 4. Total RNA was extracted from tissues with TRIzol reagent (Invitrogen, CA, USA), and miRNAs were obtained using the mirVana miRNA isolation Kit (Ambion, Austin, TX) according to the manufacturer's instructions. The quality and quantity of the RNA samples were assessed by standard electrophoresis and spectrophotometer methods. miRNA microarray analysis was performed by Biotechnology Corporation (Shanghai, China). Microarrays utilized Affymetrix microRNA chip technology and optimized RNA hybridization probes with a detection limit of <100 attomole that was cross referenced to the Sanger miRBase V 20.0 after normalization. The data from the microarray was collected and analyzed in accordance to the Microarray Experiment (MIAME) guidelines.

### TaqMan qRT-PCR detection of mature miRNA expression

Taqman MicroRNA Assays were performed as described previously [43]. Total miRNA from cells or tissues was extracted by using the mirVana miRNA Isolation Kit (Ambion, Austin, TX) according to the manufacturer's instructions. cDNA was synthesized from 5 ng of total RNA by using the Taqman miRNA reverse transcription kit (Applied Biosystems, Foster City, CA), and the expression levels of miR-129-3p were quantified by using miRNA-specific TaqMan MiRNA Assay Kit (Applied Biosystems). qRT-PCR was performed by using the Applied Biosystems 7500 Sequence Detection system. The sequences of primers are shown in Supplementary Table 5. The expression of miRNA was defined based on the threshold cycle (Ct), and relative expression levels were calculated as 2^−[(Ct of miRNA)-(Ct of U6)]^ after normalization with reference to expression of U6 small nuclear RNA.

### Real-time qRT-PCR detection of Aurora-A mRNA expression

The qRT-PCR analysis of Aurora-A mRNA was performed as described before [44]. Primers for qRT-PCR were designed using the IDT primer quest programme and were produced by MWG (Ebersberg, Germany). GAPDH was used as an internal standard. The sequences of primers were as follows: Aurora-A: forward, 5′-*AATGCCCTGTCTTACTGTCATTC*-3′ and reverse, 5′-*TCCAGAGATCCACCTTC-*

*TCATC*-3′; GAPDH: forward, 5′-*GACTCATGACCACAGTCCATGC*-3′ and reverse: 5′-*AGAGGCAGGGATGATGTTCTG*-3′. The relative transcript amount of the target gene, calculated using standard curves of serial RNA dilutions, was normalized to that of GAPDH.

### Methylation-specific PCR (MSP) and bisulfite sequencing PCR (BSP)

MSP and BSP Assays were performed as described previously [45]. Genomic DNA from tissues and cell lines was isolated with Easypure Genomic DNA kit (TransGen Biotech, Beijing, China). Genomic DNA was treated with EZ DNA Methylation-Gold™ Kit (Zymo Research Corporation, CA, USA) according to the manufacturer's instructions, and analyzed by MSP, collected the BSP product for sequencing. The PCR amplification was performed with HotStar Taq Polymerase (Qiagen, Germany), and the primer sequences were listed in Supplementary Table S3. For quantitative methylation analyses we used bisulfite pyrosequencing of miR-129-2 CpG promoter. Mean methylation level of analyzed CpG sites was used for the further analyses.

### Transfection of plasmid vectors and oligonucleotides

All microRNA mimics and inhibitors were purchased from GenePharma (Shanghai, China). Short hairpin RNA (shRNA) specifically targeting human Aurora-A (GenBank no. NM_003600) was designed to knockdown Aurora-A expression. The shRNA sequences targeting Aurora-A and negative control shRNA are listed in Supplementary Table 5. All the above sequences were inserted into the BglII and HindIII enzyme sites of pSilencer4.1-CMVneo vector, respectively. The recombinant plasmids were named pSil/shAurora-A and pSil/shcontrol, respectively. The recombinant vectors were confirmed by the digestion analysis of restriction endonuclease, and all the constructed plasmids were confirmed by DNA sequencing. The plasmid vector (pMD18/Auro) expressing open-reading frame of Aurora-A was purchased from Sino Biological Inc (Beijing, P.R. China). The cell transfection was performed in opti-MEM with the transfection reagent Lipofectamine™ 2000 (Invitrogen, CA, USA) following the manufacturer's instructions. For stable transfection, the cell lines transfected with pSil/shAurora-A (or pSil/shcontrol) vector were stably selected with G418 (400 mg/mL) 48h later after transfection, and individual clones were isolated and maintained in a medium containing G418 (100 mg/mL).

### Western blotting assay

Cell protein lysates were separated in 10% SDS polyacrylamide gels, electrophoretically transferred to polyvinylidene difluoride membranes (Roche), then detected with anti-Aurora-A, E-cadherin, N-cadherin, β-catenin, Vimentin, phosphorylated Akt (473), total Akt, phosphorylated p38-MAPK, total p38-MAPK and MMP-2 proteins. Protein loading was estimated using mouse anti-GAPDH monoclonal antibody. Lab Works™ Image Acquisition and Analysis Software (UVP, Upland, CA, USA) were used to quantify band intensities. Antibodies were purchased from Univ-bio Inc (Shanghai, China).

### Immunofluorescence staining assay

Immunofluorescence staining assay was performed following the standard protocol. Briefly, the transfected cells were fixed with 4% paraformaldehyde, permeabilized in 0.5% Triton X-100, and blocked with 10% goat serum. The cells were then incubated overnight with specific primary antibodies. After washing with PBS, the cells were incubated with fluorescence-conjugated secondary antibody for 1 h. The slides were then washed with PBS and mounted with mounting medium containing Anti-fade reagent and 4,6-diamidino-2- phenylindole. The cells were examined under a fluorescent microscope. The indirect immunofluorescence analysis was valued and performed by IPWIN60.

### Immunohistochemistry assay

Resected HCC tissues were fixed in 10% formaldehyde and embedded in paraffin. Sections (4.0 μm), cut from the original paraffin blocks, were deparaffinized in xylene and rehydrated in graded alcohols and distilled water. After inhibition of endogenous peroxidase activity for 30 min with methanol containing 0.3% H_2_O_2_, the sections were blocked with 10% normal goat serum (Invitrogen, CA, USA) for 20 min and incubated overnight with rabbit anti-human-Aurora-A (diluted 1:150, Santa Cruz Biotechnology, CA) at 4°C. The sections were then incubated with biotinylated anti-rabbit IgG for 30 min at room temperature, followed by incubation with peroxidase-conjugated avidin/biotin complexes and stained with 3, 3-diaminobenzidine tetrahydrochloride (DAB). Finally, the sections were counter-stained with hematoxylin. Normal rabbit serum was used as a negative control for the staining reactions.

### Wound healing assay

Cell migration was measured using a wound healing assay. In brief, cells were seeded in 12-well plates and cultured to confluence. Wounds of 1.0 mm width were created with a plastic scriber, and cells were washed and incubated in a serum-free medium. 48 h after wounding, cultures were fixed and observed under a microscope. A minimum of five randomly chosen areas were measured and the distance of cell migration to the wound area was determined.

### *In vitro* transwell assay

Cell invasiveness *in vitro* was reflected by the ability of cell to transmigrate a layer of extracellularmatrix in Biocoat Matrigel Invasion Chambers (Becton Dickinson Labware, Bedford, MA). The cells were seeded into inserts at 800 per insert in serum-free medium and then transferred to wells filled with the culture medium containing 10% FBS as a chemoattractant. After 24 h of incubation, non-invading cells on the top of the membrane were removed by scraping. Invaded cells on the bottom of the membrane were fixed, followed by staining with 0.05% crystal violet. The number of invaded cells on the membrane was then counted under a microscope. Each experiment was performed in triplicate.

### *In vivo* tumor growth and metastasis assay

All female BALB/c athymic nude mice at 5 to 6 weeks of age in this study were purchased from the Department of comparative medicine (Jinling Hospital, Nanjing, China). Exponentially growing HCC cells were split and grown in a fresh medium for one more day before harvest for inoculation. For *in vivo* tumor growth assay, a total of 5.0×10^6^ HCCLM3 or MHCC97-H cells transfected with miR-129-3p/mimics or miR-NC/mimics were suspended in 100 μL PBS and injected subcutaneously into the right side of the posterior flank of female BALB/c athymic nude mice (Department of comparative medicine, Jinling Hospital, Nanjing, China) at 5 to 6 weeks of age. Tumor growth was examined every 1 week with a vernier caliper. Tumor volumes were calculated by using the equation: V= A×B^2^/2 (mm^3^), wherein A is the largest diameter, and B is the perpendicular diameter. Tumor growth was monitered, and mice were sacrificed after 6 weeks and s.c. tumors were resected and fixed in 10% PBS. We then excised, measured the primary tumors. The tumors tissues were rapidly frozen in liquid nitrogen and stored at -80°C. For *in vivo* tumor metastasis assay, a total of 8.0×10^6^ HCCLM3 (or MHCC97-H) cells transfected with miR-129-3p/mimics (or miR-NC/mimics) or HepG2 (or BEL-7402) cells transfected with anti-miR-129-3p (or anti-miR-NC) in 100 μl of phosphate-buffered saline were subcutaneously injected into the flanks of nude mice. After 4 weeks, the subcutaneous tumors were resected and diced into 1.0 mm^3^ cubes, which were then implanted into the left lobes of the livers of the nude mice (10/group). After 10 weeks, the animals were killed, and lung or liver tumor nodules were counted. Survival tests were performed using groups of mice (n=10/group) treated as above and monitored daily until all the mice died. All animal studies were conducted in accordance with according to protocols that were approved by the Animal Care and Use Committee of Jinling Hospital (Nanjing University, Nanjing, China).

### Luciferase reporter constructs and luciferase assay

To construct a luciferase reporter vector, Aurora-A 3′-UTR fragment containing putative binding sites for miR-129-3p (2145-2165nt) was amplified by PCR and subcloned into the downstream of luciferase gene in the pLUC luciferase vector (Ambion, USA) and named pLUC/Aurora-A-3′-UTR-wt. Site-directed mutagenesis of the miR-129-3p target-site in the pLUC/Aurora-A-3′-UTR was performed using the Quick-change mutagenesis kit (Stratagene, Heidelberg, Germany) and named pLUC/Aurora-A-3′-UTR-mut, in which Aurora-A-3′-UTR-wt was used as a template. The sequences of PCR primers are listed in Supplementary Table 5. For luciferase reporter assays, HEK293T or HCCLM3 cells (3.0×10^5^) were plated in a 24-well plate and then co-transfected with 100 ng of pLUC/Aurora-A-3′-UTR-wt or pLUC/Aurora-A-3′-UTR-mut and 50 nM of using Lipofectamine 2000 (Invitrogen, USA). Forty eight hours after transfection, cells were harvested and assayed with Dual-Luciferase Reporter Assay kit (Promega, Madison, WI, USA) according to the manufacturer's instructions.

### RNA immunoprecipitation

RNA immunoprecipitation was performed as previously described [46]. Briefly, nuclei were isolated from HCCLM cells 24 hours after transfection. AGO2 was immunoprecipitated by using AGO2-specific antibodies (Abnova, clone 2E12-1C99) or 10 μg IgG1 isotype control antibody coupled to 25 μl of protein G Dynabeads (Invitrogen, CA, USA).

### Statistical analysis

Analysis was performed using SPSS 17.0 software (SPSS, Inc., Chicago, IL, USA). Experimental data were expressed as the mean±SEM. Quantitative variables for nonparametric analyses were performed using Wilcoxon text for paired and Mann-Whitney U test for unpaired analyses. The disease-free survival (DFS) and over survival (OS) curves were plotted using the Kaplan-Meier method and were evaluated for the statistical significance using a log-rank test. The significance of different variables with respect to survival was analyzed using the multivariate Cox proportional hazards model. The correlation was evaluated by Spearman's rank correlation coefficients. A *P*<0.05 was taken as statistically significant.

## SUPPLEMENTARY MATERIALS FIGURES AND TABLES


