# Supplementary material for: Methylation-associated silencing of microRNA-129-3p promotes epithelial-mesenchymal transition, invasion and metastasis of hepatocelluar cancer by targeting Aurora-A
Source: Oncotarget. 2016 Oct 25;7(47):78009–28. doi: 10.18632/oncotarget.12870 (PMC5363640; doi:10.18632/oncotarget.12870)
Supplement: Supplementary file 1 [file oncotarget-07-78009-s001.pdf]

Methylation-associated silencing of microRNA-129-3p promotes epithelial-mesenchymal transition, invasion and metastasis of hepatocelluar cancer by targeting Aurora-A

SUPPLEMENTARY FIGURES AND TABLES

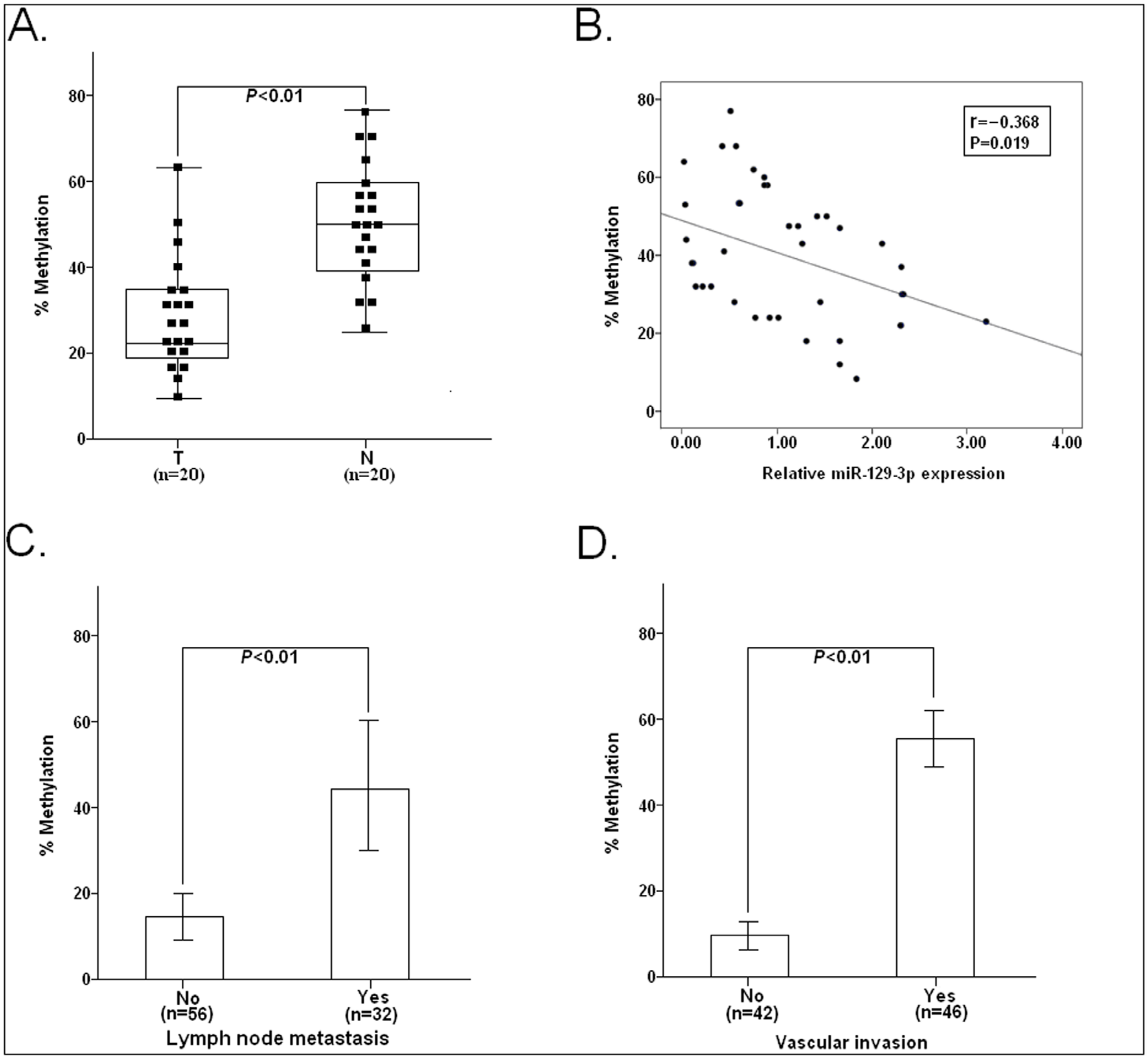

Supplementary Figure S1: Methylation-specific real-time PCR analysis of miR-129-2 promoter methylation and its correlation with miR-129-3p expression. A. Quantitative analysis of miR-129-2 promoter methylation in paired HCC and adjacent nontumor liver tissues (n=20). Statistical analyses were performed using Wilcoxon signed-rank test. T: HCC tissues; N: nontumor liver tisssues. **B.** Correlation between miR-129-2 promoter methylation and miR-129-3p expression in tissues (n=40). Statistical analyses were performed using Spearman's test. **C.** Quantitative analysis of miR-129-2 promoter methylation in HCC tissues with (n=32) or without (n=56) lymph node metastasis. Statistical analyses were performed using Wilcoxon signed-rank test. **D.** Quantitative analysis of miR-129-2 promoter methylation in HCC tissues with (n=46) or without (n=42) vascular invasion. Statistical analyses were performed using Wilcoxon signed-rank test. Each qRT-PCR experiment was performed in triplicate.

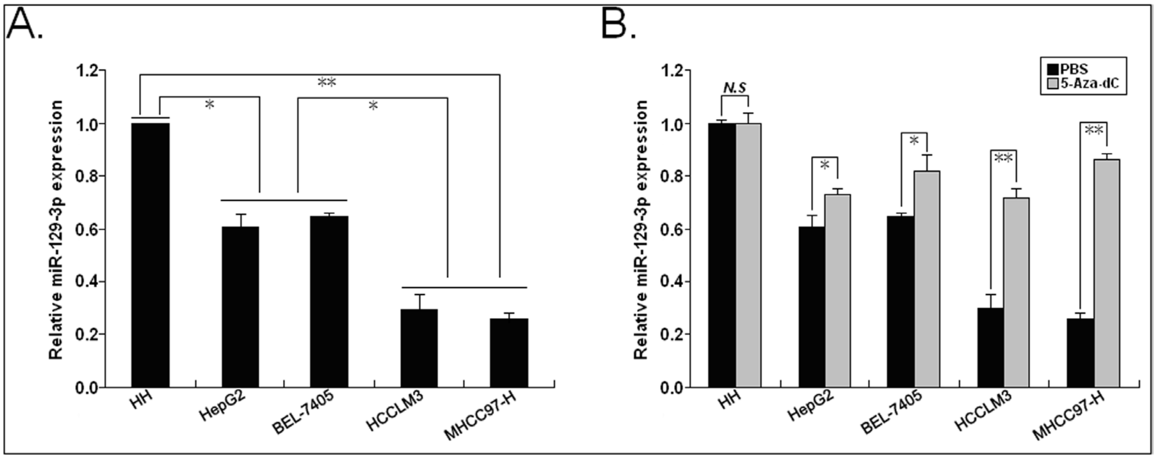

Supplementary Figure S2: Analysis of miR-129-3p expression in HCC cell lines w and its correlation with epigenetics. **A.** HCC cell lines with different metastatic potentials and a normal human hepatocyte cell line (HH). Expression of miR-129-3p was determined by qRT-PCR. **B.** The expression level of miR-129-3p in HCC cell lines with 5-Aza-dC treatment (10.0 μmol/L) was determined by qRT-PCR. Each assay was performed in triplicate. *N.S*, *P*>0.05 and **P*<0.05; ***P*<0.01.

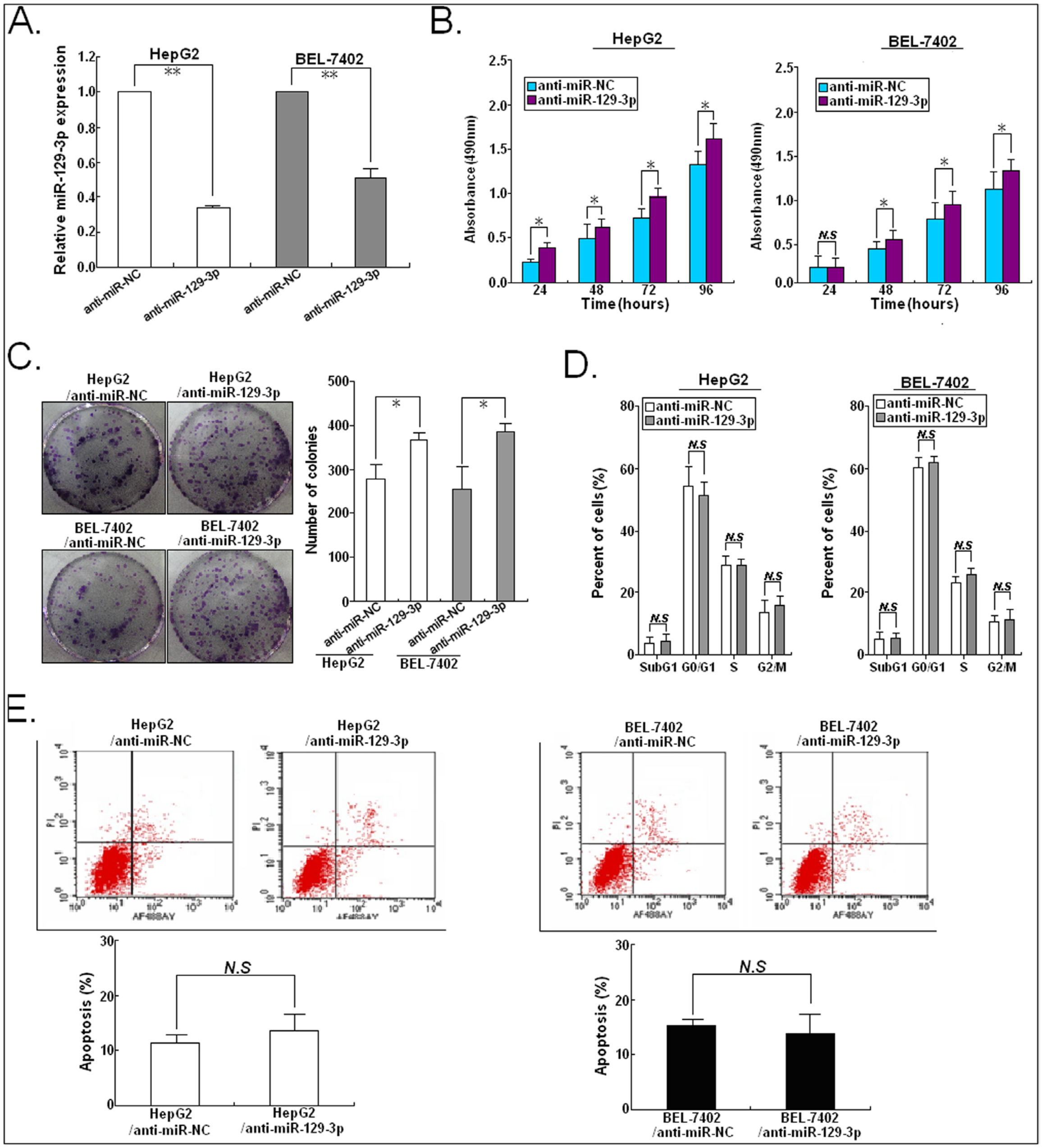

Supplementary Figure S3: Effects of miR-129-3p upregulation on *in vitro* growth, cell cycle, apoptosis and *in vivo* tumorigenecity of HCC cells. A. qRT-PCR detection of miR-129-3p expression in HCCLM3 or MHCC97-H cells transfected with miR-NC/mimics or miR-129-3p/mimics, respectively. **B.** MTT analysis of growth in HCCLM3 or MHCC97-H cells transfected with miR-NC/mimics or miR-129-3p/mimics, respectively. **C.** The colony formation of HCCLM3 or MHCC97-H cells transfected with miR-NC/mimics or miR-129-3p/mimics, repectively. **D.** Flow cytometric detection of cell cycle in HCCLM3 or MHCC97-H cells transfected with miR-NC/mimics or miR-129-3p/mimics, respectively. **E.** Flow cytometric detection of apoptosis in HCCLM3 or MHCC97-H cells transfected with miR-NC/mimics or miR-129-3p/mimics, respectively. **F.** Effects of miR-129-3p on in *vivo* tumor growth of HCC cells. (a) Tumor growth curves of subcutaneous implantation mouse models of HCC were shown. (b) Tumor volumes in the orthotopic implantation models at week 6 are shown. (c) qRT-PCR detection of miR-129-3p expression in tumors developed from HCCLM3 or MHCC97-H cells transfected with miR-NC/mimics or miR-129-3p/mimics, respectively. U6 was used as an internal control. Each experiment was performed in triplicate. *N.S*, *P*>0.05 and **P*<0.05; ***P*<0.01.

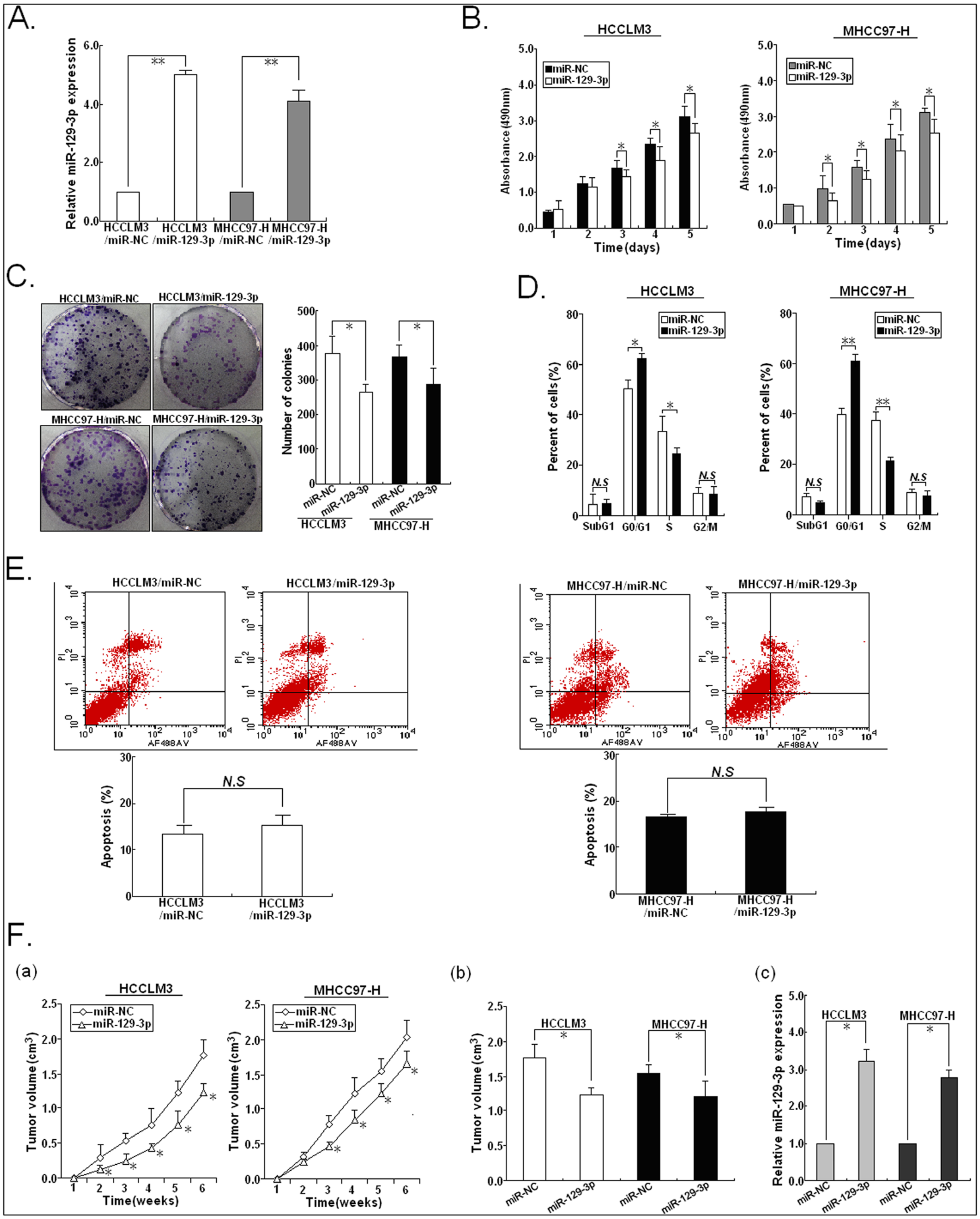

Supplementary Figure S4: Effects of miR-129-3p downregulation on *in vitro* growth, cell cycle and apoptosis of HCC cells. A. qRT-PCR detection of miR-129-3p expression in HepG2 and BEL-7402 cells transfected with anti-miR-129-3p or anti-miR-NC, respectively. U6 was used as an internal control. **B.** MTT analysis of growth in HepG2 and BEL-7402 cells transfected with anti-miR-129-3p or anti-miR-NC, respectively. **C.** The colony formation of HepG2 and BEL-7402 cells transfected with anti-miR-129-3p or anti-miR-NC, respectively. **D.** Flow cytometric detection of cell cycle in HepG2 and BEL-7402 cells transfected with anti-miR-129-3p or anti-miR-NC, respectively. Each experiment was performed in triplicate. *N.S*, $P>0.05$ and $*P<0.05$; $**P<0.01$.

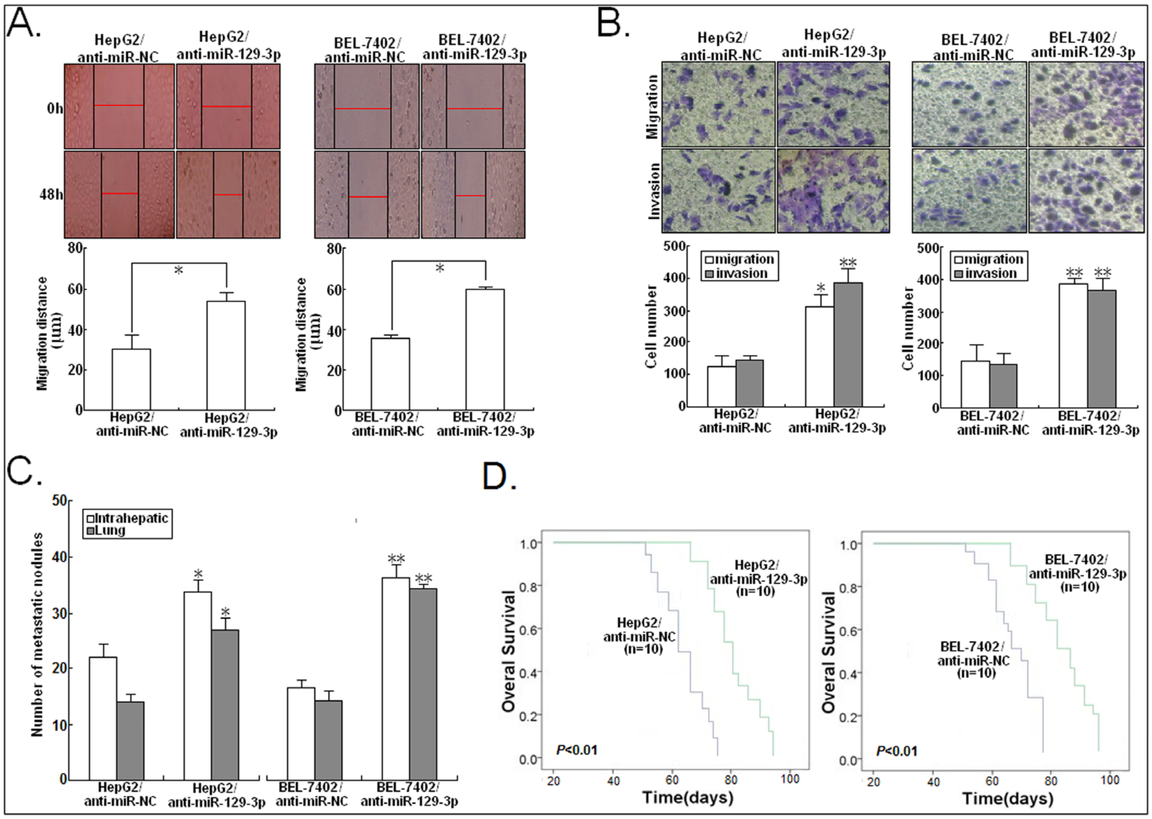

Supplementary Figure S5: Downregulation of miR-129-3p promotes *in vitro* and *in vivo* migration and invasion of HCC cells. **A.** Wound healing assay. A confluent monolayer of anti-miR-NC or anti-miR-129-3p-transfected HepG2 or BEL-7402 cells was wounded. Photographs were taken immediately (0 h) and at 48 h after wounding, quantification of wound closure was done. The data present the mean distance of cell migration to the wound area at 48 h after wounding in three independent wound sites per group. **B.** Transwell migration and invasion assay of anti-miR-NC or anti-miR-129-3p-transfected HepG2 or BEL-7402 cells. Cells in six random fields of view at 100× magnification were counted and expressed as the average number of cells per field of view. **C.** Hematoxylin and eosin staining of intrahepatic and lung metastatic tumor nodules formed from anti-miR-NC or anti-miR-129-3p-transfected HepG2 or BEL-7402 cells (n=10/group). The numbers of metastatic nodules in each nude mice were counted and statistically analyzed. **D.** The OS time of different groups of nude mice transplanted with anti-miR-NC or anti-miR-129-3p-transfected HepG2 or BEL-7402 cells (n=10/group). The survival data were compared with the log-rank test. **P*<0.05; ***P*<0.01.

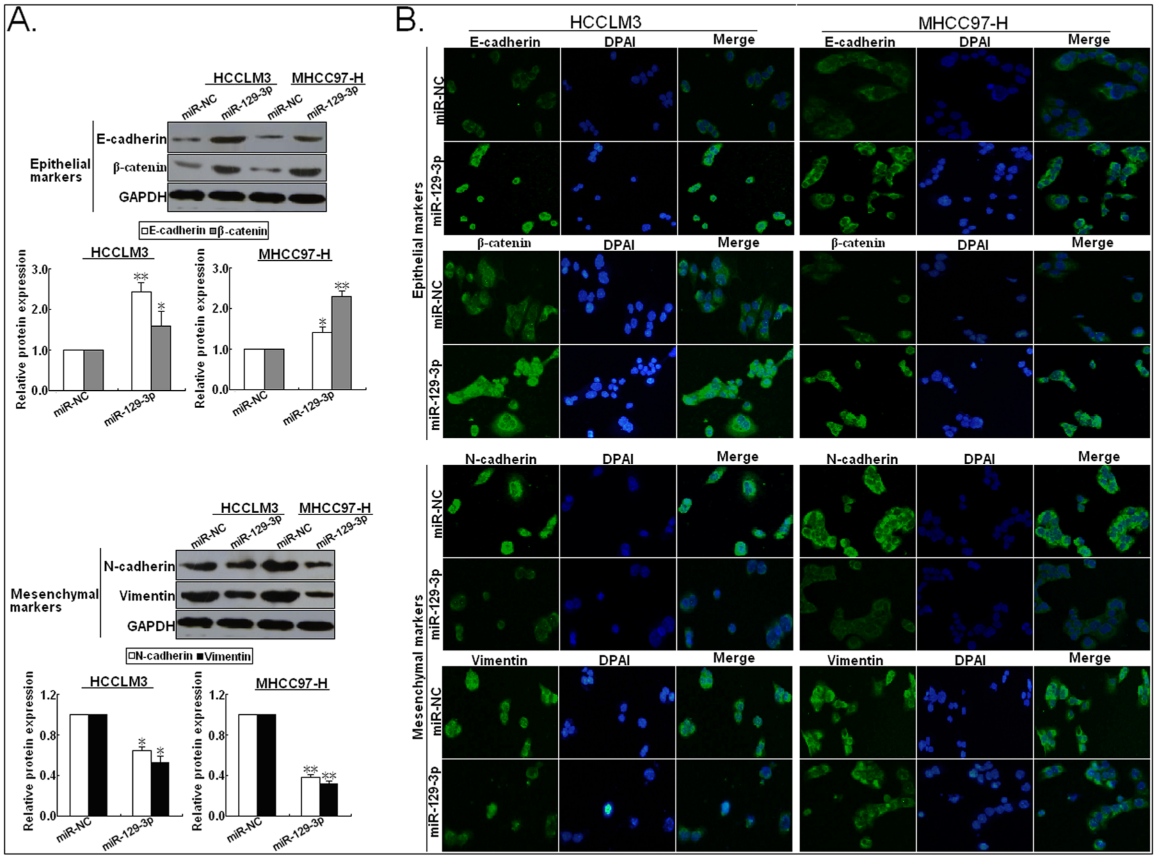

Supplementary Figure S6: Downregulation of miR-129-3p promotes EMT in HCC cells (low initial metastatic potential). **A.** Western blotting and **B.** immunofluorescence staining assays indicated that the decreased expression of epithelial markers (E-cadherin and β-catenin) and the increased expression of mesenchymal markers (N-cadherin and Vimentin) could be obviously observed in anti-miR-129-3p-transfected HCCLM3 or MHCC97-H cells, compared with anti-miR-129-3p-transfected cells. GAPDH was used as an internal control. Each experiment was performed in triplicate. $^{*}P<0.05$; $^{**}P<0.01$.

Supplementary Table S1: Correlation between miR-129-3p and clinicopathological variables of HCC patients

Variables	Relative miR-129-3p expression		*P*-value
	Low (n=52)	High (n=36)	
Gender			0.152
Female	33	28	
Male	19	8	
Age (years)			0.372
<55	21	18	
≥55	31	18	
Family HCC history			0.560
No	33	25	
Yes	19	11	
Alchohol intake			0.771
No	39	26	
Yes	13	10	
Tumor diameter (cm)			0.432
≤5.0	40	25	
>5.0	12	11	
Liver function			0.082
Child-Pugh A	28	26	
Child-Pugh B	24	10	
AFP (ng/L)			0.063
≤400	34	30	
>400	18	6	
TNM stage			0.001*
I	5	14	
II	22	14	
III	25	8	
Lymph node metastasis			0.022*
No	28	28	
Yes	24	8	
Vascular invasion			0.036*
No	20	22	
Yes	32	14	
Edmondson grade			0.053
I	14	15	
II	15	14	
III	23	7	
Recurrence			0.001*
No	2	10	
Yes	50	26	

NOTE: AFP, alpha-fetoprotein; TNM, tumor-node-metastasis. *P<0.05.

Supplementary Table S2: Univariate and multivariate Cox regression analyses of DFS in 88 HCC patients

Clinicopathological variables	Univariate analysis		Multivariate analysis	
	HR (95% CI)	*P*-value	HR (95% CI)	*P*-value
Gender (Female/Male)	1.064 (0.598-2.927)	0.874	一	一
Age (≥55 years/<55 years)	1.699 (0.819-3.525)	0.154	一	一
Family HCC history (Yes/No)	0.870 (0.398-1.901)	0.727	一	一
Alchohol intake (Yes/No)	0.489 (0.187-1.276)	0.144	一	一
Tumor diameter (>5.0 cm/≤5.0 cm)	1.802 (0.721-4.500)	0.208	一	一
Liver function (Child-Pugh B/A)	0.759 (0.268-2.148)	0.602	一	一
AFP (>400ng/L/≤400ng/L)	1.026 (0.427-2.466)	0.953	一	一
Edmondson grade (III/I+II)	1.126 (0.888-3.963)	0.489	一	一
TNM stage (III/I+II)	3.744 (2.918-4.836)	0.002*	2.924 (1.864-3.514)	0.001*
Lymph node metastasis (Yes/No)	5.119 (1.511-7.337)	0.009*	3.464 (1.383-8.673)	0.008*
Vascluar invasion (Yes/No)	1.715 (1.454-2.269)	0.047*	2.622 (0.566-2.948)	0.114
MiR-129-3p expression (Low/High)	3.647 (1.441-5.231)	0.006*	2.913 (1.254-6.770)	0.013*

Note: HR: hazard ratio; 95% CI: 95% confidence interval; *statistically significant difference.

Supplementary Table S3: Univariate and multivariate Cox regression analyses of OS in 88 HCC patients

Clinicopathological variables	Univariate analysis		Multivariate analysis	
	HR (95% CI)	*P*-value	HR (95% CI)	*P*-value
Gender (Female/Male)	1.323 (0.495-2.287)	0.489	—	—
Age (≥55 years/<55 years)	1.553 (0.758-3.184)	0.229	—	—
Family HCC history (Yes/No)	1.362 (0.620-2.989)	0.441	—	—
Alchohol intake (Yes/No)	0.634 (0.495-2.287)	0.327	—	—
Tumor diameter (>5.0 cm/≤5.0 cm)	1.313 (0.576-2.997)	0.517	—	—
Liver function (Child-Pugh B/A)	0.849 (0.318-2.263)	0.602	—	—
AFP (>400ng/L/≤400ng/L)	1.209 (0.489-2.991)	0.681	—	—
Edmondson grade (III/I+II)	1.522 (0.784-3.840)	0.457	—	—
TNM stage (III/I+II)	2.954 (1.615-3.040)	0.001*	2.924 (1.864-3.514)	0.001*
Lymph node metastasis (Yes/No)	3.111 (1.959-4.096)	0.039*	3.464 (1.383-8.673)	0.008*
Vascluar invasion (Yes/No)	1.643 (1.296-1.395)	0.004*	0.685 (0.328-1.430)	0.114
MiR-129-3p expression (Low/High)	4.207 (1.606-5.018)	0.003*	2.947 (1.522-3.239)	0.005*

Note: HR: hazard ratio; 95% CI: 95% confidence interval; *statistically significant difference.

Supplementary Table S4: Characteristics of HCC patients used for microRNA microarray detection

Case	Age (year)	Famlily HCC history	Gender	Alcohol intake	HBV infection	Tumor diameter (cm)	TNM stage	Lymph Node metastasis	Vascular invasion	Edmondson grade
HCC-L1	52	Y	M	N	N	2.0	II	N	N	I
HCC-L2	65	N	F	N	Y	1.8	I	N	N	II
HCC-L3	44	N	M	N	Y	1.2	II	N	N	II
HCC-L4	38	Y	M	N	Y	1.5	II	N	N	III
HCC-NL1	41	Y	F	N	N	3.6	III	Y	Y	II
HCC-NL2	47	Y	M	Y	Y	3.7	III	Y	Y	III
HCC-NL3	58	N	F	N	N	2.5	III	Y	N	III
HCC-NL4	53	N	M	Y	Y	2.4	III	Y	Y	III

L: with lymph node metastasis; NL: without lymph node metastasis; N: no; Y: yes;

Supplementary Table S5: Primers used in this study

Name	Primer sequence
Primers for qRT-PCR	
miRNA Universal R	*TGGTGTCGTGGAGTCG*
miR-129-3p-F	5'-*ACACTCCAGCTGGGAAGCCCTTACCCCAA* -3'
miR-129-3p-RT	5'-*CTCAACTGGTGTCGTGGAGTCGGCAATTCA GTTGAGATGCTTTT*-3'
U6-F	5'-*CTCGCTTCGGCAGCACA*-3'
U6-R	5'-*AACGCTTCACGAATTTGCGT*-3'
Aurora-A-F	5 -*AATGCCCTGTCT-TACTGTCATTC*-3'
Aurora-A-R	5'-*TCCAGAGATCCACCTTCTCATC*-3'
GAPDH-F	5'-*GACTCATGACCACAGTCCATGC*-3'
GAPDH-R	5'-*AGAGGCAGGGATGATGTTCTG*-3'
Oligo sequence	
Hairpin of shAurora-A-F	5'-*GATCCATGCCCTGTCTTAACTGTCATTCAAG-AGATGACAGTAAGACAGGGCATAGA*-3'
Hairpin of shcontrol-F	5'-*GATCCAAGCTGAAGTACAACCTTCTTCAAG-AGAGAAGGTTGTACTTCAGCTTAGA*-3'
Primers for Aurora-A 3'-UTR cloning	
Wild type 3'-UTR-F	5'-*GGCTCGAGCATGTGTCTCAGAGCTGTTAAGGGCTTA*-3'
Wild type 3'-UTR-R	5'-*GCGGATCC AGACATAGATACTTATTTATTT*-3'
Mutant type 3'-UTR-F	*5'-GGCTCGAGCATGTGTCTCAGAGCTGTUGGGCUUA*-3'
Mutant type 3'-UTR-R	5'-*GCGGATCC AGACATAGATACTTATTTATTT*-3'
